# Non-invasive digital etching of van der Waals semiconductors

**DOI:** 10.1038/s41467-022-29447-6

**Published:** 2022-04-05

**Authors:** Jian Zhou, Chunchen Zhang, Li Shi, Xiaoqing Chen, Tae Soo Kim, Minseung Gyeon, Jian Chen, Jinlan Wang, Linwei Yu, Xinran Wang, Kibum Kang, Emanuele Orgiu, Paolo Samorì, Kenji Watanabe, Takashi Taniguchi, Kazuhito Tsukagoshi, Peng Wang, Yi Shi, Songlin Li

**Affiliations:** 1 National Laboratory of Solid-State Microstructures and Collaborative Innovation Center of Advanced Microstructures, Nanjing University, Nanjing, China; 2 School of Electronic Science and Engineering, Nanjing University, Nanjing, China; 3 College of Engineering and Applied Sciences and Jiangsu Key Laboratory of Artificial Functional Materials, Nanjing University, Nanjing, China; 4 Department of Physics, Southeast University, Nanjing, China; 5 Department of Materials Science and Engineering, Korea Advanced Institute of Science and Technology, Daejeon, Republic of Korea; 6 Institut national de la recherche scientifique, Centre Énergie Matériaux Télécommunications, 1650 Blvd. Lionel-Boulet, J3X 1S2 Varennes, QC Canada; 7 University of Strasbourg, CNRS, ISIS UMR 7006, 8 allée Gaspard Monge, F-67000 Strasbourg, France; 8 WPI-MANA, National Institute for Materials Science, Tsukuba, 305-0044 Ibaraki, Japan; 9 Department of Physics, University of Warwick, CV4 7AL Coventry, UK

**Keywords:** Two-dimensional materials, Electronic devices

## Abstract

The capability to finely tailor material thickness with simultaneous atomic precision and non-invasivity would be useful for constructing quantum platforms and post-Moore microelectronics. However, it remains challenging to attain synchronized controls over tailoring selectivity and precision. Here we report a protocol that allows for non-invasive and atomically digital etching of van der Waals transition-metal dichalcogenides through selective alloying via low-temperature thermal diffusion and subsequent wet etching. The mechanism of selective alloying between sacrifice metal atoms and defective or pristine dichalcogenides is analyzed with high-resolution scanning transmission electron microscopy. Also, the non-invasive nature and atomic level precision of our etching technique are corroborated by consistent spectral, crystallographic, and electrical characterization measurements. The low-temperature charge mobility of as-etched MoS_2_ reaches up to 1200 cm^2^ V^−1^s^−1^, comparable to that of exfoliated pristine counterparts. The entire protocol represents a highly precise and non-invasive tailoring route for material manipulation.

## Abstract (web summary)

Here, the authors exploit a non-invasive layer-bylayer etching technique to fabricate electronic devices based on 2D transition metal dichalcogenides with controlled thickness and transport properties comparable to those of exfoliated flakes.

## Introduction

Modern science and technology have benefited vastly from the ever-increasing capability of fine control on material dimensions. For instance, in two-dimensional (2D) van der Waals materials, the reduced dimensionality by approaching the atomic thickness can result in major changes in fundamental physical characteristics such as the density of states, band structures^1,2^, crystal electrostatic fields^3,4^ and even lattice symmetries, which allows for emerged couplings of material parameters, such as electron wavefunction radii and magnetic interaction lengths. This provides a platform for exploring intriguing physical phenomena such as Dirac fermions^2^, valley chirality^5^, Moiré superlattices^3,4^, and thickness-sensitive magnetism^6,7^. On the other hand, the continuous downscaling in modern microelectronics has brought the industry to technology nodes of several nanometers^8^. In the near future, a finer control up to atomic levels will be soon required^9^. In this context, atomically thin 2D semiconductors, in particular monolayer transition-metal dichalcogenides (TMDCs) which exhibit ideal surface flatness and sizeable carrier mobility, are regarded as promising channel materials in the post-silicon era^9–11^. For achieving large-area atomic channels, the most straightforward but yet challenging route is to bottom-up grow homogeneous monolayer wafers directly, while it also represents a possible route to tailor the pristinely inhomogeneous few-layer wafers into homogeneous ones through selective top-down etching.

For both the fundamental and applied purposes, it is highly anticipated that the original crystallinity, thus the intrinsic physical properties of the materials, are preserved after material processing. In this regard, a protocol enabling simultaneously non-invasive and digital etching of TMDCs, that is precise layer-by-layer removal of the topmost monolayer while keeping the underlying intact, represents a keen anticipation and, at the same time, a big challenge. Previous approaches developed for atomic etching of van der Waals TMDCs, including plasma bombardment^12–14^, laser treatment^15,16^, and thermal oxidation^17^, are all proven invasive to materials because they generally exert less selective etching effects on the layers to be removed and preserved, thus resulting in degraded electronic performance. To date, the techniques that allow for non-invasive etching of the 2D van der Waals crystals are still highly sought after.

Here we report a soft protocol for non-invasive digital etching of van der Waals TMDCs by exploiting the selective thermal diffusion of a sacrificial metal into assigned TMDC layers and subsequent removal of the surfacial alloy layer. Synergic strategies including surface defect engineering and low-temperature annealing were devised to strictly limit the etching depth to a monolayer at each etching cycle. The nature of layer-by-layer digital etching was confirmed by aberration-corrected high-resolution scanning transmission electron microscopy (HR-STEM), atomic force microscope (AFM), secondary harmonic generation (SHG), and Raman spectroscopy. Both the crystallographic and electrical characterizations suggest that the high lattice quality and intrinsic transport performance are preserved in the as-etched samples, implying the non-invasivity of the protocol. Besides the non-invasivity and atomic-level precision, the strategy also features important advantages including multiple processability, universality for different TMDC materials, and CMOS compatibility. The underlying physics for the selective thermal diffusion between nearly perfect pristine and defective van der Waals lattices is also extensively studied. The research results well address a grand etching challenge confronted in material science and open a door to thoroughly exploit the van der Waals TMDC crystals for quantum and electronic purposes.

## Results and discussion

### Rationale for defect engineering

**Fig. 1:**
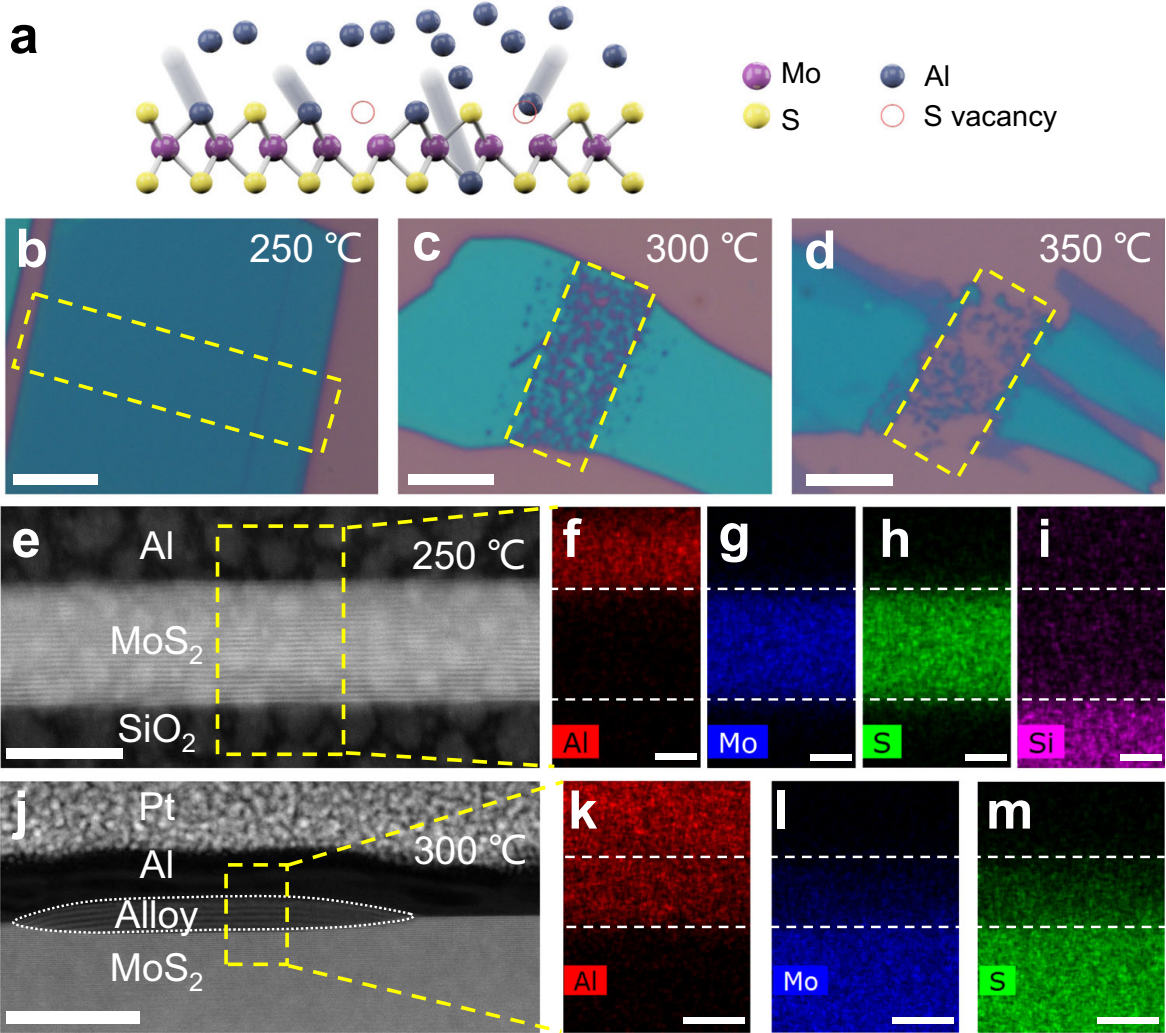
Uncontrollable thermal diffusion of Al atoms into pristine MoS_2_ lattices. **a** Schematic diagram for the thermal diffusion process, where randomly distributed surfacial defects serve as the starting sites to guide the diffusion inside the MoS_2_ lattices. **b**–**d** Typical optical images of three acid-washed MoS_2_ samples after one-hour thermal diffusion of Al at *T*_a_ = 250, 300, and 350 °C, respectively. The dashed yellow rectangles outline the areas of locally deposited sacrificial metal Al strips. Scale bars, 5 μm. **e**–**i** Cross-sectional HAADF image and corresponding elemental mappings for a typical Al/MoS_2_/SiO_2_ stack annealed at 250 °C. The dashed white lines in **f**–**i** highlight the two sharp interfaces of MoS_2_. Scale bars in **e** and **f**–**i** are 20 and 6 nm, respectively. **j**–**m** Cross-sectional HAADF image and corresponding elemental mappings for a typical Al/MoS_2_ stack annealed at 300 °C. The dotted white line in **j** shows the alloy region between Al and MoS_2_. The dashed white lines in **k**–**m** highlights the interfaces of Al/alloy and alloy/MoS_2_. Scale bars in **j** and **k**–**m** are 40 and 6 nm, respectively.

Our protocol for the non-invasive digital layer-by-layer etching mainly comprises the following steps. First, metal Al sacrificial layers are deposited selectively onto local defect-engineered TMDC areas to be thinned. Then, controlled thermal diffusion of Al into TMDCs is achieved by tuning the temperature and duration in the subsequent step of thermal annealing. Finally, the layers of Al and its alloy with TMDCs are wet etched with acid or basic solutions. We emphasize that, without appropriate defect engineering in the first step, a simple implementation of such protocol could cause uncontrollable Al diffusion and thus random etching into underlying layers in our preliminary attempts, as depicted in Fig. 1a. In circumstance that no artificial defects are produced, we found that the thermal diffusion of Al into TMDCs still relies on the randomly distributed raw vacancies present in the TMDC lattices^18^, which result in disordered diffusion inside the van der Waals materials, because they can reduce local diffusion energy barriers and serve as the starting sites to guide the disordered diffusion, as shown in Fig. 1c.

### Uncontrollable thermal diffusion of Al into pristine MoS_2_

In the preliminary experiments where no artificial defect engineering was performed, we first investigated the thermal diffusion mechanism of Al into freshly mechanically exfoliated MoS_2_, which is proven to be the defect-assisted diffusion process as mentioned above, by simply exploring the dependence of diffusion rate on annealing temperature (*T*_a_). Figure 1b–d shows the optical images of three MoS_2_ samples washed with acid, after 1 h thermal diffusion of Al at *T*_a_ = 250, 300, and 350 °C, respectively. With increasing *T*_a_ the corrosion pits become deeper and spatially more extended, as indicated by the dashed rectangles. Indubitably, *T*_a_ is an important parameter to promote the Al atoms to overcome the diffusion barriers within MoS_2_ and, normally following the Arrhenius relationship, to determine the diffusion rate and overall etching depth^18^. Noteworthy, at low *T*_a_ (up to 250 °C) no appreciable traces of Al diffusion and corrosion pits are observed, suggesting a negligible diffusion of Al into the pristine (nearly perfect) MoS_2_ lattices. For samples annealed at intermediate *T*_a_ (300 °C), a mixture of corrosion pits and unetched islands are monitored in Fig. 1c, indicating that the thermal diffusion of Al into pristine MoS_2_ lattice is a poorly uniform process, mainly guided by the disordered raw lattice vacancies present in pristine samples. Although the pristine MoS_2_ can be thinned at this temperature, the thickness reduction features a modest control.

Cross-sectional high-angle annular dark-field imaging (HAADF) and energy-dispersive X-ray spectroscopy (EDS) by HR-STEM made it possible to monitor the boundaries between layers to further unveil the interlayer diffusion behavior at different *T*_a_s. Figure 1e–i shows the typical HR-STEM images and corresponding EDS elemental mappings for the Al/MoS_2_/SiO_2_ stacks annealed at 250 °C. They exhibit two sharp interfaces for the encapsulated MoS_2_ layers, validating the negligible diffusion of the upper Al or the underlying SiO_2_ into the encapsulated MoS_2_ layers at such a low *T*_a_. For comparison, the images for the stack annealed at 300 °C are displayed in Fig. 1j–m.

The HAADF image displayed in Fig. 1j is informative. First, it shows that the interlayer diffusion of Al and MoS_2_ indeed occurs at this elevated *T*_a_﻿, resulting in the alloying and delamination of local MoS_2_ up to 6 layers in the central area. The Al/MoS_2_ alloy area indicated by dotted lines resembles a surface bubble. Second, the thermal diffusion of metal atoms into the van der Waals materials appears as a rather ordered process along the vertical direction that tends to terminate at the van der Waals gaps and gives rise to a clear diffusion boundary in the central area. Thus, it is expected that the breadth of the diffusion depth can be further finely controlled within a monolayer at a low *T*_a_ below 300 °C. Third, a mixed combination of alloyed and unreacted areas (sharp boundaries), with roughly equal probabilities, are observed in the extended HR-STEM images (Supplementary Fig. 2a), suggesting the diffusion mechanism in pristine MoS_2_ as follows. Given the low energy of formation of sulfur vacancies and the trend of sulfur loss at elevated temperatures^19–21^, it is deduced that the random Al diffusion is associated with the disordered sulfur vacancies rawly present and/or thermally created in MoS_2_ lattices. They reduce the diffusion barriers of local areas and act as permeation paths for external atoms, resulting in the disordered Al diffusion through the underlying MoS_2_ layers^18,22^.

### Principle of controllable diffusion via defect engineering

To circumvent the issue of random and excessive Al diffusion, we devised a “selective diffusion” strategy aimed at producing uniform diffusion sites and, at the same time, spatially confining diffusion depth within the thickness of one monolayer. The basic idea is to predefine surfacial lattice vacancies distributed uniformly in the topmost layer before thermal diffusion, which is expected to reduce the diffusion barrier of the topmost layer only and produce a vast difference in the diffusion rates, which increase exponentially by lowing diffusion barrier, of Al atoms between the defective topmost and pristine underlying TMDC layers. Theoretical studies revealed that Ar plasma irradiation can produce various single- and multiple-atom vacancies (e.g., S, Mo, and MoS_6_) into MoS_2_ lattices^23^. Hence, by applying a controlled short duration of Ar plasma irradiation and introducing a limited low density of vacancies in the surfaces, the topmost layer can be tailored to exhibit much larger diffusion rates than the underlying layers. Accordingly, the topmost and underlying layers would exhibit selective overall etching rates, which is beneficial for selectively etching the topmost layer while minimizing the negative corrosive impact on the underling layers. In addition, we try to minimize *T*_a_ as low as 250 °C, which can ensure regular diffusion rates for Al into defective topmost layer but negligible rates into pristine underlying layers, as proven in Fig. 1b.

Further theoretical calculations indicate that the diffusion barrier for Al via sulfur vacancies into a defective MoS_2_ lattice is about 90 meV, being 8 times lower than into a pristine crystal (Supplementary Note 5). According to the Arrhenius relationship, the eight-fold variation in diffusion barrier corresponds to a huge difference of 10^6^ in thermal diffusion coefficients between the defective and pristine lattices at 250 °C, constituting the rationale behind the selective etching.

### Layer-by-layer digital etching

**Fig. 2:**
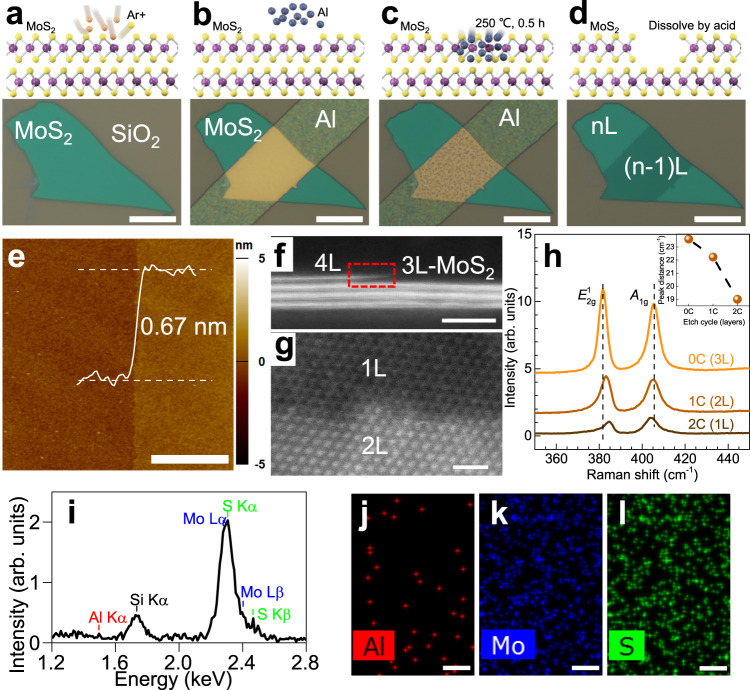
Non-invasive digital etching technique and corresponding characterization. **a**–**d** Schematic processing flow for the controllable digital etching. Scale bar: 10 μm. **a** Controlled Ar plasma irradiation to produce uniformly distributed sulfur vacancies in the topmost MoS_2_ layer. **b** Local deposition of sacrificial metal Al strips on MoS_2_. **c** Thermal annealing at 250 °C for 0.5 h to facilitate the diffusion of Al into MoS_2_. **d** Dissolving Al and related alloy by hydrochloric acid. The topmost MoS_2_ layer is removed finally, where nL denotes the number of layers. Digitally etched monolayer steps (0.67 nm) between the pristine and etched areas as revealed by **e** AFM, **f** cross-sectional, and **g** top-view atomic images. Scale bar in **e**: 3 μm. Scale bar in **f**, **g**: 2 nm. **h** Raman spectra for pristine (0C) and etched local MoS_2_ areas for one cycle (1C) and two cycles (2C). Inset: Peak distance between the ${E}_{2{{{{{\rm{g}}}}}}}^{1}$ and *A*_1g_ modes versus etching cycles (i.e., number of MoS_2_ layers). **i** Accumulated energy dispersive spectrum from the as-etched monolayer to estimate the content of Al residues. **j**–**l** Typical EDS elemental mappings for the Al, Mo, and S elements, respectively. The trace of Al residues is below the instrumental uncertainty of 5%. Scale bars in **j**–**l**: 1 nm.

With the “selective diffusion” strategy in mind, we then perform the devised non-invasive digital etching protocol on defective engineered MoS_2_ samples. Figure 2a–d illustrates the processing flow and corresponding optical images at each step for the soft etching technique. Initially, a low-energy Ar plasma irradiation is applied onto exfoliated MoS_2_ layers to introduce vacancies into the topmost MoS_2_ layers (Fig. 2a). The irradiation energy and duration are carefully controlled to ensure that only the topmost sulfur atoms are affected without damaging the underlying layers (Supplementary Notes 8 and 9). Then, a 10 nm metal Al strip is deposited onto MoS_2_ (Fig. 2b), followed by a diffusion process at 250 °C for 0.5 h (Fig. 2c). At such a low *T*_a_, the Al atoms diffuse mainly through the defective topmost layer, leaving the underlying layers almost intact. Lastly, the Al strip and its alloy with the topmost MoS_2_ layer are dissolved in hydrochloric acid and, consequently, a monolayer of MoS_2_ is removed (Fig. 2d).

A critical requirement for the digital etching technique is to precisely control the etching depth down to one monolayer. To check the etching depth and surface flatness of the as-etched area, we performed AFM measurements on the as-etched samples in order to determine the thickness change and surface topography. As shown in Fig. 2e, after one-cycle (1C) processing, the step height between the pristine (0C) and etched areas is about 0.67 nm, which is consistent with the theoretical value of the thickness of a MoS_2_ monolayer, being 0.615 nm^24^, and verifies the nature of layer-by-layer etching of this technique. Besides, the root mean square roughness of the etched area is estimated to be 0.38 nm, which is comparable to that of the pristine area ~0.43 nm, proving the excellent surface quality of the as-etched area. Cross-sectional and top-view images recorded by HR-STEM also confirmed the nature of layer-by-layer tailoring of this technique. In Fig. 2f a sharp monolayer step produced by etching can be clearly seen in the cross-sectional image taken at the trilayer/tetralayer (3L/4L) step, while in Fig. 2g a clear contrast between the monolayer (1L) and bilayer (2L) areas can be observed in the top-view image taken for an etched 2L sample. Moreover, the HR-STEM images can be used to accurately evaluate the lateral resolution of our etching technique, owing to unwanted lateral diffusion. After analysis on the edge profiles of the alloy areas, we conclude that the lateral resolution at above etching condition is better than 1.5 ± 0.3 nm. Detailed discussion can be found in Supplementary Note 4.

To further confirm the nature of digital layer-by-layer etching, Raman spectra were also recorded for a twice-etched 3L sample, which contains 0C, 1C, and 2C etched areas, as shown in Fig. 2h. It is well known that the thickness of few-layer TMDCs can be determined from the distance of their characteristic Raman modes^25,26^. With the decrease in MoS_2_ thickness, the distance between the ${E}_{2{{{{{\rm{g}}}}}}}^{1}$ and *A*_1g_ modes is decreased accordingly. As shown in the inset of Fig. 2h, the distance values for the three contrastive areas amount to 23.6, 22.2, and 19 cm^−1^, which correspond to the thickness values of 3L, 2L, and 1L, respectively. These consecutive numbers of thickness provide unambiguous evidence for the accurate thickness control featuring monolayer precision and the way as digital tailoring in the process.

Since this etching method finds its roots in the diffusion and removal of Al atoms, which may reside on the surfaces of the as-etched materials, it is also important to check the lattice quality and trace of Al residues atop the materials. To cast light onto this issue, we also performed atomically resolved top-view STEM and EDS elemental analyses for as-etched MoS_2_ monolayers, as shown in Supplementary Fig. 12 and Fig. 2i–l. In a series of atomic STEM images, we performed a statistical analysis to estimate the gross vacancy density of about (1.3 ± 0.6) × 10^13^ cm^−2^, without ruling out the extra bombardment effect from the electron irradiation during STEM imaging. This value is comparable to (1.2 ± 0.4) × 10^13^ cm^−2^, the value reported in high-quality exfoliated sheets^27^, indicating the negligible invasiveness of our etching technique. In Fig. 2i, the EDS spectrum collected over a large area reveals that the trace of Al residues, located at 1.49 keV, is below the instrumental uncertainty of 5%. Elemental mappings for Al, Mo, and S also indicate there are no detectable Al signals (Fig. 2j), as compared with the strong Mo and S signals (Fig. 2k, l). Note that the sparse bright pixels in Fig. 2j arise likely from the “background” chamber contamination and random noise of the imaging system. Detailed discussion can be found in Supplementary Fig. 13. Besides, XPS analysis (Supplementary Fig. 14) also supports the conclusion that the trace of Al residues is negligible.

### Alternative method for defect engineering

As far as surface defect engineering is concerned, besides the Ar plasma irradiation, there are also various methods to introduce vacancies into the topmost layer, such as thermal decomposition. We tested this method by pre-annealing for 1 h the freshly exfoliated MoS_2_ samples at 400 °C, a temperature near the critical point for the escape of sulfurs on the surface, before Al deposition. Under such a thermal decomposition condition, the density of sulfur vacancies is greatly enhanced at the MoS_2_ surface, although the surface exhibits no remarkable change under optical microscope (Supplementary Fig. 15a). As a result, after the low-temperature annealing together with a sacrificial Al strip at 250 °C for 0.5 h (Supplementary Fig. 15b) and acid wash, the topmost layer is clearly removed, as shown in Supplementary Fig. 15c. We note that this process would not take place without the artificial defects introduced by proper pre-annealing. Hence, the thermal decomposition of the surface sulfur atoms via pre-annealing also represents an appropriate defect engineering strategy that results in the layer-by-layer etching, as it follows the concept of selective etching.

### Multiple digital etching

**Fig. 3:**
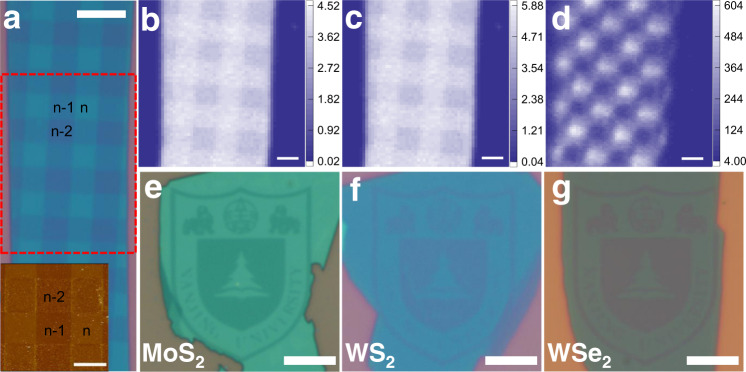
Fabricating complex patterns on different TMDCs. **a** A checkerboard-like motif on MoS_2_ defined with two stripy etching cycles along the vertical and horizontal directions. Scale bar: 6 μm. Inset: the AFM image scanned from a local area of the checkerboard-like motif. The label *n*, *n* − 1, and *n* − 2 represent the numbers of local MoS_2_ areas. Scale bar, 3 μm. **b**–**d** Raman and SHG mapping for the local area denoted by the red dashed rectangle in (**a**). Raman mappings were acquired by calculating the area ratio of the **b** ${E}_{2{{{{{\rm{g}}}}}}}^{1}$ and **c** *A*_1g_ modes of MoS_2_ to the 520 cm^−1^ mode of Si. The bright and blue areas in the SHG mapping in panel **d** denote the local areas with odd and even numbers of MoS_2_ layers. Scale bars in **b**–**d**, 3 μm. **e**–**g** The patterned logos for Nanjing University on exfoliated MoS_2_, WS_2_, and WSe_2_, respectively. Scale bars, 6 μm.

For both lab and industrial applications, complex patterning and multiple etching steps are highly sought after. Next, we demonstrated the possibility of applying this procedure repeatedly to construct complex patterns when combined with electron beam lithography (EBL). Figure 3a shows the optical image of a checkerboard-like pattern on a MoS_2_ sheet that is defined by alternatively applying the etching procedures (defect engineering via Ar plasma irradiation) along the vertical and horizontal directions. According to such patterning motifs, local areas with the three consecutive numbers of layers *n*, *n* − 1, and *n* − 2 are constructed, where *n* is the number of unetched layers. The success in this patterning characterized by consecutive numbers of layers, can be clearly seen by means of optical, AFM, and Raman characterizations. According to previous research^25^, the ratio of the Raman modes of MoS_2_ to Si substrate can be used to discern the thickness information of MoS_2_ samples with the number of layers up to 10. In Fig. 3b, c, we mapped the area ratios of the Raman modes of ${E}_{2{{{{{\rm{g}}}}}}}^{1}$ and *A*_1g_ to Si, respectively, where the checkerboard-like pattern is clearly seen.

Second-harmonic generation (SHG) response^28^, being a sensitive probe for the lattice symmetry of thin TMDCs, was also employed to verify the variation of number of layers. It has been shown that MoS_2_ sheets with odd numbers of layers display a significant SHG response while those with even numbers of layers exhibit almost zero response. Figure 3d displays the SHG mapping for the sample; it exhibits alternative light and dark motifs along the vertical and horizontal directions, in good accordance with the checkerboard-like pattern. All these characterizations indicate that our soft etching technique is atomically precise in achieving uniform layer-by-layer etching, with no obvious residues after each etching step.

We also exploited this method to various TMDCs with more delicate patterns to demonstrate its universal applicability. Figure 3e–g portrays the optical images of patterned logos of Nanjing University on three different TMDCs: MoS_2_, WS_2_, and WSe_2_, respectively. The nearly identical logo patterns imply that the concept of selective etching is likely applicable for various TMDCs and van der Waals materials, if they can be treated in appropriate conditions to ensure required defect density in topmost layers, annealing temperature, duration of thermal diffusion, and concentration of wash solution, etc. Raman and photoluminescent spectra were also employed to characterize the pristine and etched WS_2_ and WSe_2_ sheets, as shown in Supplementary Fig. 16. All data support the validity of our method.

### Electronic evidence for non-invasive etching

**Fig. 4:**
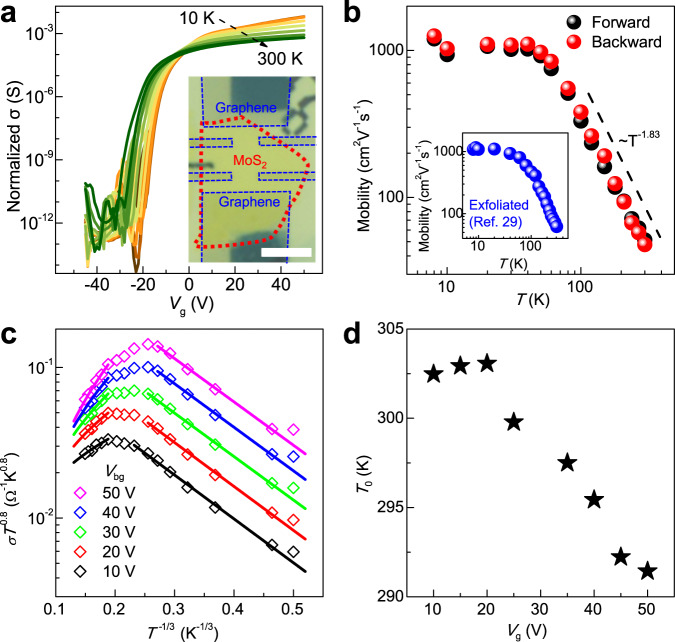
Electrical measurement for a typical as-etched 4L MoS_2_ encapsulated by ultraclean h-BN dielectrics. **a** Transfer characteristics for the as-etched 4L MoS_2_ at various different *T* values of 10, 30, 50, 80, 120, 180, 240, and 300 K. Inset: Optical image for the BN/Graphene/MoS_2_/BN structure where graphene (labeled by dashed blue lines) is used as the electrodes for MoS_2_ channel (dashed red lines) in the standard Hall geometry. Scale bar, 4 μm. **b** *T*-dependent field-effect mobility. At *T* > 60 K, the mobility follows the power law $\mu \propto {T}^{-\gamma }$ with *γ* = 1.83. The black dashed line (${ \sim T}^{-1.83}$) is a guide to the eyes. Inset: $\mu -T$ curve of a typical mechanically exfoliated multi-layer MoS_2_. Adapted with permission from ref. ^29^. Copyright 2015 American Chemical Society. Our etched sample exhibits comparable performance to exfoliated counterparts. **c** *T*-dependent normalized conductance at various gate voltages. At low *T* regime the carrier transport can be described by Mott VRH model, while it turns into the band-like transport behavior at high *T* regime. **d** Values of characteristic temperature (${T}_{0}$) estimated from the Mott VRH model at low *T* regime versus gate voltage. The values are rather small, indicating that the disorder due to Al residues is insignificant.

In order to evaluate the effect of extra lattice defects and Al residues on the overall electrical quality, cryogenic electrical characterizations were carried out on as-etched MoS_2_ layers. After etching, the remained MoS_2_ samples were contacted by multiple graphene electrodes and were encapsulated by two ultraclean hexagonal boron nitride (h-BN) dielectrics. Standard Hall geometry was adopted to estimate its intrinsic electrical performance, as shown in the inset of Fig. 4a. Figure 4a shows typical transfer curves (four-terminal conductivity *σ* versus ${V}_{{{{{{\rm{g}}}}}}}$) for an FET channel made from as-etched 4L MoS_2_ at different temperature (*T*) values from 10 to 300 K. The device exhibits high current on/off ratios of ${10}^{9}{-10}^{11}$, suggesting that the semiconducting nature is well preserved in the as-etched sample. The curves intersect around ${V}_{{{{{{\rm{g}}}}}}}$ ~ 0, indicating the emergence of metal–insulator transition upon modulating carrier concentration. This behavior is consistent with that reported in high-quality samples^29^ and confirms again the preservation of high crystallinity after etching.

In Fig. 4b, we plot intrinsic carrier mobility ($\mu$) versus *T* to further discern the effect of carrier scattering from Al residues. At low *T*, $\mu$ becomes saturated at ~1200 cm^2^ V^−1^s^−1^. This value is consistent with exfoliated samples^29^. At high *T*, $\mu$ follows a power law with *T* ($\propto {T}^{-\gamma }$ with $\gamma \sim$ 1.83) in the log–log plot. Theoretical studies on pure phonon scattering predicts an exponent $\gamma$ ~ 1.69 and ~2.6 for 1L and bulk MoS_2_, respectively^30^. However, the addition of Coulomb impurity scattering will degrade $\mu$ in the entire *T* regime and, as a result, reduce $\gamma$ largely. For instance, $\gamma$ ~ 0.62–0.72 were reported in SiO_2_ supported monolayer MoS_2_ samples^31,32^. The $\gamma$ value in our device is reasonably within the range of theoretical prediction, suggesting the dominancy of scattering from lattice phonons and the negligibility of effect from Coulomb impurities such as Al residues. The entire $\mu -T$ trend of the as-etched samples is comparable to that of h-BN encapsulated exfoliated counterparts^29^ (inset of Fig. 4b), thus it can be inferred that the acid wash can remove nearly all the Al residues and provide a clean and fresh surface for the as-etched samples.

Finally, we analyze the transport mechanism of the etched layer based on the variable *T* transport characteristics. As previously reported^31,33^, due to the atomic thickness, the surface disorders, such as the adsorbates atop and substrate charges underneath, tend to cause Anderson localization, especially at low carrier densities. Even in the case of highly crystalline few-layer MoS_2_, the presence of a high density of localized states in the band-gap region can lead to variable-range hopping (VRH) carrier transport behavior. To gain further insight into the transport mechanism, we plot in Fig. 4c*σ* versus *T* in terms of the modified 2D Mott VRH equation^31,33^

1 $$\sigma =A\cdot {T}^{m}\cdot {{{{{\rm{exp }}}}}}\left(-{\left(\frac{{T}_{0}}{T}\right)}^{1/3}\right),$$

where *A* is a constant, *m* is the coefficient normally adopted as −0.8 for 2D TMDCs, and ${T}_{0}$ is the characteristic temperature. The parameter ${T}_{0}$ is related to the localization length $\xi (E)$ which can be used to qualitatively estimate the degree of disorder, and can be described as

2 $${T}_{0}=\frac{13.8}{{k}_{{{{{{\rm{B}}}}}}}{\xi }^{2}\left(E\right)D\left(E\right)},$$

where ${k}_{{{{{{\rm{B}}}}}}}$ is the Boltzmann constant, and $D({{{{{\rm{E}}}}}})$ is the typical density of trap states from interfacial residues. The fitted values of ${T}_{0}$ is plotted versus ${V}_{{{{{{\rm{g}}}}}}}$ in the inset of Fig. 4d. ${T}_{0}$ keeps fixed around 300 K, which is 1–3 orders lower in magnitude than the values reported in literature^31,33^, indicating the large localization length and insignificant disorders induced by Al residues. This feature corroborates further the insignificance of the extrinsic disorders introduced by Al residues.

In summary, we have devised a selective etching protocol enabling the atomically precise and non-invasive layer-by-layer etching of 2D van der Waals materials, based on thermal diffusion and subsequent alloy dissolution of a local metal sacrificial layer. Such an etching protocol addresses the challenge of non-invasive tailoring material thickness with an atomic precision. This technique is universal, being applicable to different van der Waals TMDC materials, as it only requires the van der Waals materials to be stable in acid or basic solutions. The realization of atomic precision in the etching process and the high crystalline quality of the as-etched layers (i.e., surface morphology and electronic transport) is demonstrated by a series of characterization means including HR-STEM, AFM, Raman, SHG response, and *T* variable electronic measurement. Our protocol for in situ layer-by-layer etching is easily up-scalable, thus suitable for both lab and industrial applications.

## Methods

### Pristine crystals

The MoS_2_ crystal was selected from natural minerals, WS_2_ purchased from 2D Semiconductors Inc., and WSe_2_ synthesized by chemical vapor transport. All thin TMDC layers used in the experiment to be etched were mechanically exfoliated from corresponding crystals and transferred to Si substrates with a 90 or 285 nm SiO_2_ capping layer.

### Etching procedure

At first, the exfoliated TMDC layers were spin-coated with a layer of A4 PMMA electron resist and the areas to be etched were then exposed by EBL, transferred to an inductively coupled plasma (ICP) chamber for Ar plasma irradiation. The ICP chamber was initially pumped to a base vacuum of 8 × 10^−3^ Pa and was then filled with a constant Ar flow of 10 sccm till the working pressure of 0.5 Pa. The TMDC samples were then irradiated in Ar plasma at an ICP generator power of 30 W and an idle CCP biasing power for 30 s. The CCP power was set in the idle mode in all experiment to minimize plasma energy and the attacking depth. The energy of Ar plasma is about 60–70 eV. Subsequently, the Al sacrifice strips were deposited on the TMDC samples by thermal evaporation at a rate of 0.7–1.2 Å/s. A thickness of 10 nm was employed to ensure complete coverage of the areas to be etched. After liftoff, the TMDC samples partially covered with the Al strips were then annealed under a 2 torr nitrogen atmosphere. The annealing recipe for the layer-by-layer etching is 250 °C for 0.5 h, while the recipes for the control experiments are 250, 300, and 350 °C for 1 h to determine the diffusion depths of Al into MoS_2_ at different temperatures. Finally, the annealed samples were immersed into dilute hydrochloric acid (~16%) for 0.5 h, to completely remove the unreacted Al and its alloy with TMDCs, and then were rinsed in deionized water multiple times.

### Raman and SHG mapping

Raman spectra were acquired at an excitation wavelength of 488 nm with a power of 22 mW. The integration time was 5 s when collecting spectra for individual points and was reduced to 1 s in the mapping mode to avoid loss of focus during the long-time collection. SHG was excited by a 1550 nm fiber laser with an 80 MHz repetition rate. The laser power is set at 100 mW and the integration time used is 100 ms.

### HR-STEM imaging

The cross-sectional STEM samples were fabricated by a lift-out method using focused ion beam technique (FEI Helios 600i dual-beam system). The STEM lamellas were thinned down to a thickness below 100 nm using a beam current of 0.79 nA at 30 kV, followed by gentle milling with a beam current of 72 pA at 2 kV. The top-view STEM samples for atomic imaging were prepared by direct transfer of as-etched MoS_2_ monolayers from Si substrates onto 300-mesh copper STEM grids through polymer PMMA as transfer medium. HR-STEM HAADF images and EDS mapping were acquired on a double aberration-corrected FEI Titan Cubed G2 60-300 S/TEM at 60 kV equipped with a Super-X EDS detector.

### Device fabrication and characterization

The mechanically exfoliated MoS_2_ samples were firstly transferred by Poly-(propylene carbonate) (PPC) films from Si/SiO_2_ substrates onto ultraclean h-BN flakes exfoliated on other Si/SiO_2_ substrates, followed by our non-invasive etching method to remove the top layer. Then, few-layer graphene Hall electrodes that were pre-etched were picked-up by top h-BN flakes supported with PPC/PDMS bilayers. Subsequently, the PPC layers were heated till softened to release the h-BN/graphene bilayers as the top encapsulator/electrode onto the as-etched MoS_2_/bottom h-BN bilayers to form the target h-BN/graphene/MoS_2_/h-BN structures. Afterwards, electrode vias were selectively opened on the top h-BN layers by standard EBL and CF_4_ plasma exposure to expose the graphene Hall electrodes. Finally, steps of EBL and metallization of 10 nm Ni/50 nm Au were carried out to wire out the graphene Hall electrodes for electrical characterization. Electrical measurements were performed in vacuum at ${10}^{-5}$ Torr using a probe station (CRX-6.5 K, Lake Shore) and two Keithley 2636B sourcemeters. The values of four-terminal field-effect mobility of as-etched MoS_2_ channels were calculated by using the equation $\mu =\frac{1}{{C}_{{{{{{\rm{ox}}}}}}}}\cdot \frac{{{{{{\rm{d}}}}}}\sigma }{{{{{{{{\rm{d}}}}}}V}}_{{{{{{\rm{g}}}}}}}}$, where the *σ* is the normalized conductance of the channels, ${C}_{{{{{{\rm{ox}}}}}}}$ is the capacitance of the bilayer dielectric structure of h-BN (~10 nm) and SiO_2_ (90 or 285 nm).

## Supplementary information

Supplementary Information

## Data Availability

The data that support the plots within this paper and other finding of this study are available from the corresponding authors on reasonable request.

## References

[1] Mak KF, Lee C, Hone J, Shan J, Heinz TF (2010). Atomically thin MoS_2_: A new direct-gap semiconductor. Phys. Rev. Lett..

[2] Crossno J (2016). Observation of the Dirac fluid and the breakdown of the Wiedemann–Franz law in graphene. Science.

[3] Cao Y (2018). Correlated insulator behaviour at half-filling in magic-angle graphene superlattices. Nature.

[4] Chen G (2019). Evidence of a gate-tunable Mott insulator in a trilayer graphene Moiré superlattice. Nat. Phys..

[5] Schaibley JR (2016). Valleytronics in 2D materials. Nat. Rev. Mater..

[6] Gong C (2017). Discovery of intrinsic ferromagnetism in two-dimensional van der Waals crystals. Nature.

[7] Huang B (2017). Layer-dependent ferromagnetism in a van der Waals crystal down to the monolayer limit. Nature.

[8] Razavieh A, Zeitzoff P, Nowak EJ (2019). Challenges and limitations of CMOS scaling for FinFET and beyond architectures. IEEE Trans. Nanotechnol..

[9] Chen M-L (2020). A FinFET with one atomic layer channel. Nat. Commun..

[10] Li S-L, Tsukagoshi K, Orgiu E, Samori P (2016). Charge transport and mobility engineering in two-dimensional transition metal chalcogenide semiconductors. Chem. Soc. Rev..

[11] Liu Y (2021). Promises and prospects of two-dimensional transistors. Nature.

[12] Kim KS (2019). Ultrasensitive MoS_2_ photodetector by serial nano-bridge multi-heterojunction. Nat. Commun..

[13] Kim KS (2017). Atomic layer etching mechanism of MoS_2_ for nanodevices. ACS Appl. Mater. Interfaces.

[14] Liu Y (2013). Layer-by-layer thinning of MoS_2_ by plasma. ACS Nano.

[15] Sunamura K, Page TR, Yoshida K, Yano T-A, Hayamizu Y (2016). Laser-induced electrochemical thinning of MoS_2_. J. Mater. Chem. C.

[16] Castellanos-Gomez A (2012). Laser-thinning of MoS_2_: On demand generation of a single-layer semiconductor. Nano Lett..

[17] Wu J (2013). Layer thinning and etching of mechanically exfoliated MoS_2_ nanosheets by thermal annealing in air. Small.

[18] Mehrer, H. *Diffusion in Solids: Fundamentals, Methods, Materials, Diffusion-controlled Processes* Vol. 155 (Springer, 2007).

[19] Nan H (2014). Strong photoluminescence enhancement of MoS_2_ through defect engineering and oxygen bonding. ACS Nano.

[20] Donarelli M, Bisti F, Perrozzi F, Ottaviano L (2013). Tunable sulfur desorption in exfoliated MoS_2_ by means of thermal annealing in ultra-high vacuum. Chem. Phys. Lett..

[21] Liu M (2017). Temperature-triggered sulfur vacancy evolution in monolayer MoS_2_/graphene heterostructures. Small.

[22] Kasap, S. & Capper, P. (eds). *Springer Handbook of Electronic and Photonic Materials* (Springer, 2017).

[23] Bae S (2017). Defect-induced vibration modes of Ar^+^-irradiated MoS_2_. Phys. Rev. Appl.

[24] Wilson J, Yoffe A (1969). The transition metal dichalcogenides discussion and interpretation of the observed optical, electrical and structural properties. Adv. Phys..

[25] Li S-L (2012). Quantitative Raman spectrum and reliable thickness identification for atomic layers on insulating substrates. ACS Nano.

[26] Li H (2012). From bulk to monolayer MoS_2_: Evolution of Raman scattering. Adv. Funct. Mater..

[27] Hong J (2015). Exploring atomic defects in molybdenum disulfide monolayers. Nat. Commun..

[28] Li Y (2013). Probing symmetry properties of few-layer MoS_2_ and h-BN by optical second-harmonic generation. Nano Lett..

[29] Liu Y (2015). Toward barrier free contact to molybdenum disulfide using graphene electrodes. Nano Lett..

[30] Kaasbjerg K, Thygesen KS, Jacobsen KW (2012). Phonon-limited mobility in *n*-type single-layer MoS_2_ from first principles. Phys. Rev. B.

[31] Jariwala D (2013). Band-like transport in high mobility unencapsulated single-layer MoS_2_ transistors. Appl. Phys. Lett..

[32] Yu Z (2014). Towards intrinsic charge transport in monolayer molybdenum disulfide by defect and interface engineering. Nat. Commun..

[33] Ghatak S, Pal AN, Ghosh A (2011). Nature of electronic states in atomically thin MoS_2_ field-effect transistors. ACS Nano.

