# Supplementary material for: Non-invasive digital etching of van der Waals semiconductors
Source: Nat Commun. 2022 Apr 5;13:1844. doi: 10.1038/s41467-022-29447-6 (PMC8983769; doi:10.1038/s41467-022-29447-6)
Supplement: Supplementary file 1 — Supplementary Information [file 41467_2022_29447_MOESM1_ESM.pdf]

Supplementary Information

Non-invasive digital etching of van der Waals semiconductors

Jian Zhou[1,2,†], Chunchen Zhang[1,3,†], Li Shi[4], Xiaoqing Chen[1,2], Tae-Soo Kim[5], Minseung Gyeon[5], Jian Chen[1,2], Jinlan Wang[4], Linwei Yu[1,2], Xinran Wang[1,2],, Kibum Kang[5], Emanuele Orgiu[6], Paolo Samorì[7,*], Kenji Watanabe[8], Takashi Taniguchi[8], Kazuhito Tsukagoshi[8], Peng Wang[1,3,9,*] Yi Shi[1,2,*], Songlin Li[1,2,*]

[1] National Laboratory of Solid-State Microstructures and Collaborative Innovation Center of Advanced Microstructures, Nanjing University, Nanjing, China
[2] School of Electronic Science and Engineering, Nanjing University, Nanjing, China
[3] College of Engineering and Applied Sciences and Jiangsu Key Laboratory of Artificial Functional Materials, Nanjing University, Nanjing, China
[4] Department of Physics, Southeast University, Nanjing, China
[5] Department of Materials Science and Engineering, Korea Advanced Institute of Science and Technology, Republic of Korea
[6] Institut national de la recherche scientifique, Centre Énergie Matériaux Télécommunications, 1650 Blvd. Lionel-Boulet, J3X 1S2 Varennes, Canada
[7] University of Strasbourg, CNRS, ISIS UMR 7006, 8 allée Gaspard Monge, F-67000 Strasbourg, France
[8] National Institute for Materials Science, Tsukuba, Ibaraki 305-0044, Japan
[9] Department of Physics, University of Warwick, Coventry CV4 7AL, UK
[†] These authors contributed equally: Jian Zhou, and Chunchen Zhang.
[*] Correspondence should be addressed to S.L. (sli@nju.edu.cn) or to P.S. (samori@unistra.fr) or to P.W. (wangpeng@nju.edu.cn) or to Y.S. (yshi@nju.edu.cn).

Table of Content

1. PREPARATION OF CROSS-SECTIONAL STEM SPECIMENS 2
2. UNCONTROLLABLE DIFFUSION BETWEEN AL AND PRISTINE MoS_2 2
3. FLUCTUATION OF DIFFUSION DEPTH IN PRISTINE MoS_2 3
4. ESTIMATION OF LATERAL RESOLUTION IN DIGITAL ETCHING 3
5. THEORETICAL ESTIMATION OF DIFFUSION ENERGY 4
6. MECHANISM OF THERMAL DIFFUSION 5
7. OPTIMIZING PRETREATMENT CONDITIONS 6
8. ETCHING YIELD UNDER OPTIMIZED PRETREATMENT CONDITIONS 7
9. EVIDENCE FOR SUB-MONOLAYER ATTACKING DEPTH OF AR PLASMA 8
10. SOLUTIONS FOR WET ETCHING 8
11. PREPARATION OF TOP-VIEW STEM GRIDS 9
12. STATISTICS ON VACANCY DENSITY OF AS-ETCHED MONOLAYERS 9
13. ESTIMATION OF UNCERTAINTY FOR TOP-VIEW ELEMENTAL MAPPING 9
14. CHECK OF AL RESIDUES WITH XPS 10
15. AN ALTERNATIVE METHOD FOR DEFECT ENGINEERING 10
16. UNIVERSALITY OF THE ETCHING METHOD 10
17. ELECTRICAL PROPERTIES OF AS-ETCHED MoS_2 ON SiO_2/SI 11
REFERENCES 12

1. Preparation of cross-sectional STEM specimens

The cross-sectional STEM specimens were fabricated by a lift-out method using focused ion beam technique (FEI Helios 600i dual-beam system). The Al/MoS_2 stacks were firstly prepared on SiO_2/Si substrates, followed by appropriate thermal annealing. Supplementary Fig. 1**a, b** shows the optical images for typical samples after annealing at 250 and 300 °C, respectively. Before FIB milling for preparing the cross-sectional slices for STEM imaging, metal Pt was deposited onto the Al/MoS_2 stacks to protect samples from ionizing radiation during ion milling. Panel **c** and **d** show the corresponding cross-sectional slices for the overall quintuple-layer Pt/Al/MoS_2/SiO_2/Si stacks for Panel **a** and **b**, respectively. Each layer can be discerned from enlarged SEM images, as shown in Panel **e** and **f**.

2. Uncontrollable diffusion between Al and pristine MoS_2

Supplementary Fig. 2 shows an extended (~1 μm wide) cross-sectional HR-STEM image for a typical Al/MoS_2 (pristine) stack annealed at 300 °C for 1 h, which provide a chance to understand the mechanism of interlayer diffusion and alloying between the sacrificial Al and pristine MoS_2 layers. The density of surface defects on the pristine MoS_2 is believed to distribute uniformly within the lattice, since it is a freshly exfoliated sheet with high quality. Hence, the thermal diffusion of Al atoms into the MoS_2 lattice is expected to be spatially uniform without showing obvious difference along the entire cross section.

However, a mixed combination of diffused (circled by dotted write lines) and undiffused areas (showing sharp boundaries), with roughly equal distribution probabilities, are observed in the extended HR-STEM image shown in Panel **a**. Such non-uniform thermal diffusion behavior can be attributed to the formation of local defects at initial annealing stage, which facilitates subsequent Al diffusion into the defective local MoS_2 lattices. Likely, less defects are believed to be present in the undiffused areas and the relative integrity of the lattices hinders the diffusion of Al.

Panels **b** and **c** show the enlarged HAADF images for the diffused and undiffused areas, respectively. In Panel **b**, 6 layers of MoS_2 exhibiting expanded layer spacings can be clearly seen in the alloy area. Most expanded layers (the bottom 4 layers) remain parallel to each other, indicating the interlayer diffusion between Al and MoS_2 is quite an order process. At such an annealing condition (300°C, 1h), Al consumes MoS_2 up to 6 layers and the length of interlayer diffusion reaches ~10 nm. In Panel **c**, sharp layer

boundaries and no expanded MoS_2 layers are observed at the Al/MoS_2 interface in the HAADF image of the undiffused region, implying that no obvious Al/MoS_2 alloy is formed.

3. Fluctuation of diffusion depth in pristine MoS_2

For the stacks of Al and pristine MoS_2 annealed at 300 °C for 1 h, we find that there is variation in diffusion depths ranging from 3 to 6 layers in the alloy areas. Typical cross-sectional HAADF images are shown in Supplementary Fig. 3, where the diffused and delaminated MoS_2 layers are indicated by the numbers from 1 to 6, counted from the topmost to the bottom layers. The Diffusion depths are 3L, 4L, 5L, 6L from Panel **a** to **d**, respectively. The fluctuation in diffusion depth of 3-6 layer can be attributed to the overall inhomogeneity caused by multiple imperfect surfacial and crystallographic factors from involved materials, including inhomogeneously condensed absorbates on MoS_2 surfaces, non-uniform thermal stress, randomly distributed point and line defects inside MoS_2, and even the polycrystalline orientations of Al.

Hence, it would be very difficult to well control the depth of diffusion and thus etching in pristine TMDCs without defect engineering. To improve the controllability in diffusion depth, appropriate defect engineering is necessary.

4. Estimation of lateral resolution in digital etching

It is possible to estimate the length of lateral diffusion of Al directly from the profiles of the bird-beak shaped alloy edges. Supplementary Fig. 4 shows the typical profiles of the beak edges with different penetration depths, all formed at annealing condition of 300 °C, 1 h. We plot in Panel **a**, for instance, the critical diffusion terminals at the beak edge (P1, P2, P3) and the directions for Al diffusion. By assuming P0 is the starting point of diffusion, the diffusion velocities are V_{ab} and V_c along the lattice ab- and c-directions, and P1, P2, P3 are also the terminals of lateral diffusion at each layer counted from the topmost 1, 2 and 3, respectively. Indeed, all the terminals comprise the profile lines of the beak edges, which is much clearer in Panels **e** and **f** when the penetration depth is larger. The lateral distances from the terminal of the bottom layer (P3 in case of Panel **a**) to those of the upper etching layers are labelled as d_n where n = 1, 2, 3…, representing the number of upper layers counted from the topmost. For simplicity, we use d_0 to label the etching distance in the last layer, that is the lateral distance between the terminal of the bottom layer (P3 in case of Panel **a**) and the starting point of etching

(P0). Hence, the overall diffusion distance of Al at each layer can be written as d_0+d_n, where d_0 is unknown but d_n can be accurately measured from the STEM images.

Supplementary Table 1. Values of lateral distance measured from the terminal of the bottom layer to those of the upper etching layers.

Panel No.	d_1 (nm)	d_2 (nm)	d_3 (nm)	d_4 (nm)	d_5 (nm)
a	4.2	3.0	-	-	-
b	4.2	3.0	-	-	-
c	5.5	4.2	3.0	-	-
d	7.7	6.0	4.3	-	-
e	18.9	16.9	15.4	12.9	9.5
f	22.2	20.9	18.9	15.4	10.4

By taking the selective of the overall diffusion distance, we can finally obtain the individual diffusion distance (d_{n+1}-d_n, or d_n for the last layer) for each etching layer, which represents exactly the lateral diffusion distance of Al at the specific layer when Al penetrates a monolayer vertically along c direction. Thus, the lateral resolution at the topmost layer is 1.5±0.3 nm under the annealing condition of 300 °C, 1 h.

Supplementary Table 2. Values of lateral selective distance at each layer.

Panel No.	1st layer d_2-d_1 (nm)	2nd layer d_3-d_2 (nm)	3rd layer d_4-d_3 (nm)	4th layer d_5-d_4 (nm)	5th layer d_5 (nm)
a	1.3	-	-	-	-
b	1.3	-	-	-	-
c	1.3	1.3	-	-	-
d	1.7	1.7	-	-	-
e	2.0	1.5	2.5	3.5	9.5
f	1.4	2.0	3.5	5.0	10.4
**Average**	**1.5**	**1.6**	**3.0**	**4.2**	**9.9**
**Error bar**	**0.3**	**0.3**	**0.7**	**1.1**	**0.7**

It is likely that the issue of lateral diffusion of Al would arise at elevated annealing temperatures (above 300 °C) or in case of deep etching (removal of several layer at one cycle). However, we have managed to lower the annealing temperature to the safe value at 250 °C by using the defect engineering, and we etch only a monolayer at one cycle.

5. Theoretical estimation of diffusion energy

To justify the concept of selective etching and to confirm the effect of TMDC surface defects on interlayer diffusion, theoretical calculations on diffusion barriers are carried out for two types of MoS_2 lattices: defective and perfect, as shown in

Supplementary Fig. 5. The calculations are based on methods as follows. Density functional theory (DFT) calculations were performed by using the VASP package. The electron-ion interactions were evaluated by using the projector-augmented wave (PAW) pseudopotential. Exchange-correlation interactions were considered in the generalized gradient approximation (GGA) using the Perdew-Burke-Ernzerhof (PBE) and Heyd-Scuseria-Ernzerhof (HSE) methods. The van der Waals interactions were described by using the DFT+D2 scheme. The climbing image nudged elastic band (CI-NEB) method was used to find minimum energy paths.

For simplicity, we only considered the diffusion process of one Al atom. Panel **a** shows the schematic diagram for the ideal thermal diffusion process for an Al atom into a defective MoS_2 bilayer with one S vacancy on the surface. The potential curve at different coordinates of the MoS_2 lattice is schematically plotted on the right. Panels **b** and **c** show the estimated diffusion barriers for defective and perfect bilayer MoS_2 lattices, respectively. Theoretical calculations indicate that the diffusion barrier for the individual Al atom *via* sulfur vacancies into the defective MoS_2 lattice is about 90 meV (Panel **b**), corresponding to the movement of Al atom from the position A to C in the schematic potential curve in Panel **a**. The atomic configurations between Al and MoS_2 in different reaction states, i.e., initial/transition/final state (IS, TS, FS), are also given in Panel **b**. Panel **c** shows the diffusion potentials of IS and FS for Al into perfect MoS_2 bilayers, where a relatively high diffusion barrier of 750 meV is estimated. This value is more than 8 times higher than that in a defective lattice. According to the Fick's second law of diffusion, an 8-fold variation in diffusion barrier corresponds to a huge difference of 10^6 in thermal diffusion coefficients at 250 °C. Such a huge difference in diffusion coefficient constitutes the rationale behind the selective etching concept.

6. Mechanism of thermal diffusion

According to the Fick's second law of diffusion $\frac{\partial C}{\partial t} = \nabla \cdot (D \nabla C)$[1], where C is the concentration of Al atoms in alloy, t is the diffusion time of duration and D is diffusion coefficient, the behavior of thermal diffusion of Al can be well described by solving the diffusion equation above. In the diffusion theory, D depends on annealing temperature T_a following the Arrhenius relation $D = D_0 \cdot \exp(-\frac{\Delta H}{k_B T_a})$, where D_0 is a coefficient to be determined from experiment, ΔH is the activation enthalpy of diffusion, and k_B is the Boltzmann constant.

Using a continuum model for van der Waals materials (Supplementary Fig. 6), we can simply estimate the distribution of Al concentration along the diffusion direction. Since the deposited Al film (10 nm) is much thicker than Al/MoS_2 alloy region, we further assume the Al source is an infinite reservoir and its concentration keeps constant at the Al/MoS_2 interface during the whole diffusion process. By combining the boundary conditions, the Fick's diffusion law for Al distribution into MoS_2 can be simplified as $C(x,t) = C_{\mathrm{s}} \cdot \mathrm{erfc}\left(\frac{x}{2\sqrt{Dt}}\right)$, where the x is the diffusion distance along the one-dimensional flux direction (as shown in Panel **a**), C_{s} is the constant surface concentration at x = 0, and erfc( ) is the complementary error function. By substituting the exact expression of D, the function of Al concentration can be written as

$$C(x,t,\Delta H,T_a) = C_{\mathrm{s}} \cdot \mathrm{erfc}\left(\frac{x}{2\sqrt{t \cdot D_0 \cdot e^{-\frac{\Delta H}{k_B T_a}}}}\right). \quad (1)$$

By applying the experimental observation that the penetration depth is 6L for defective areas annealed at 300°C for 1h and assuming that $C(6\mathrm{L}) = 0.1\, C_{\mathrm{s}}$ (cutoff criterion), we can extract $D_0 = 4.3 \times 10^{-17}\ \mathrm{cm^2 s^{-1}}$.

Accordingly, we estimate that at 250 °C the diffusion coefficients for Al into defective and perfect MoS_2 lattices are $5.9 \times 10^{-18}\ \mathrm{cm^2 s^{-1}}$ and $2.6 \times 10^{-24}\ \mathrm{cm^2 s^{-1}}$ respectively, which features a difference of 6 orders in magnitude. Panel **b** shows the normalized concentration of Al as a function of diffusion depth for various samples and annealing conditions, with taking the concentration value $0.1\, C_{\mathrm{s}}$ as the diffusion cutoff (dashed line in Panel **b**). Within such a criterion, the diffusion depth for Al into defective MoS_2 is about 4L at the annealing condition of 250 °C, 0.5 h, while it is almost zero into perfect MoS_2. The theoretical calculations further justify the proposed concept of selective etching.

7. Optimizing pretreatment conditions

In experiment, we found that the ICP power of 30 W (CCP biasing power: idle mode) is almost the lowest parameter to excite stable Ar plasma in our facility and hence we adopted this value as the power parameter. In addition to theoretical calculation, we also cross-over tested the etching results for the surface pretreated MoS_2 sheets under different pretreatment conditions with the duration of plasma irradiation (30 W) varying from 20 to 40 s and thermal annealing T_a changing from 150 to 250 °C.

Supplementary Fig. 7 **a–i** shows the etching results that are the optical images for the tested samples before and after etching for the 9 conditions used in total. Panel **j** shows the image contrast of the etched areas with respect to nearby substrate areas, which can be used to scientifically judge the etching completeness. The criterion is set as 0.99, contrast above which standing for a completer etching process without residues.

The tendency is that samples processed under weak conditions show obvious flake remnants (Panels **a**, **b**, **d** and **g**) or light residues (Panels **c**, **e** and **h**), indicating an incomplete etching for a monolayer. In contrast, a monolayer can be completely removed under intensified conditions (Panels **f** and **i**). Overall, the parameters "30 W, 30 s and 250 °C" is concluded as an optimal pretreatment condition. The detailed conditions are listed as below.

1) Generator RF (ICP) power: 30 W (low power to minimize plasma density)
2) Biasing RF (CCP) power: 0 W (idle mode to minimize plasma energy)
3) Energy: 60–70 eV (estimated energy obtained from the RF generator)
4) Flux of Ar+: 4.5×10^{17} $cm^{-2}s^{-1}$
5) Duration of irradiation: 30 s
6) Dose of Ar^{+}: 1.4×10^{17} cm^{-2}
7) Net dose to remove S atoms: 2.2×10^{14} cm^{-2} (estimated from XPS)
8) Etching mechanism: physical bombardment

8. Etching yield under optimized pretreatment conditions

We performed XPS characterization to estimate the density of removed sulfur atoms after plasma irradiation. We cut three monolayer CVD MoS_2 samples from one wafer and exposed them in Ar plasma (ICP power: 30 W, CCP power: 0 W) under different durations from 0, 90, to 180 s. The untreated sample (duration: 0 s) is used as reference and extended durations of 90 s and 180 s are employed to increase the differences among samples.

Supplementary Fig. 8 shows the XPS spectra and corresponding fittings for the three samples, in order to extract the atomic ratios between S and Mo elements. Evidently, the intensity of the S peak located around 227 eV is reduced when the irradiation duration increases from 0 to 180 s. In panel **d**, the extracted S/Mo atomic ratio is plotted versus irradiation duration. The ratios are 1.96, 1.42 and 1.20 for samples under irradiations of 0, 90 and 180 s, respectively, indicating that the loss of S atoms is roughly proportional to plasma duration. A slight saturation emerges under high

duration, which can be attributed to the reduced probability of physical bombardment between S and Ar^+ (i.e., reduced etching yield of S atoms by Ar ions) in the highly defective samples.

By assuming a constant etching yield in the first 90 s, for the 30-s irradiation condition employed in manuscript, we estimated a S/Mo ratio of 1.78, a removal of 9.2% S atoms from the topmost layer, and an effective dose of 2.2×10^{14} cm^{-2} by Ar plasma to remove S atoms. Together with the flux of Ar+ (4.5×10^{17} $cm^{-2}s^{-1}$, ICP: 30 W, CCP: 0W), we estimated a low etching yield of ~0.16% for removal of S atoms at this weak plasma condition.

9. Evidence for sub-monolayer attacking depth of Ar plasma

To check the realistic attacking depth of Ar plasma and whether it only attacks the atoms in the topmost layer, we collected Raman spectra for pristine and irradiated monolayers (Supplementary Fig. 9), which show that the signals from lattice vibration disappear only above intensified conditions (ICP = 40 W, idle CCP, 50 s). Under the condition of 30W (ICP) and 50 s, the Raman signal still survives, indicating such a condition cannot destroy the entire lattice. Thus, under the optimized Ar plasma condition (ICP = 30 W, idle CCP, 30 s), the attacking depth into chaclogenides is within monolayer.

10. Solutions for wet etching

In order to verify the universal role of both the acid and basic solutions played for wet etching in the final processing step, we show in Supplementary Fig. 10 the results of post-etching with three various acids (sulfuric acid, phosphoric acid, oxalic acid) and one basic (Tetramethylammonium Hydroxide, TMAH) solutions. It proved that all of the four types of solutions work for the removal of Al and alloy layers. However, it deserves noting that, since the SiO_2 substrates can also be etched by strong basic solutions, they should be avoided to use in case of SiO_2 as substrates. When the supported substrates are slightly etched, the TMDC layers above the SiO_2 substrates would roll up (Panel **d**) or even be rushed away in the basic solution. Hence, basic solutions are compatible only with alkali-proof substrates.

11. Preparation of top-view STEM grids

Supplementary Fig. 11 shows typical optical images for as-etched 1L MoS_2 (Panel **a**) before and (Panels **b–d**) after transferring onto the STEM grids. Panels **b–d** show the images from low to high magnification ratios. The scale bars are 50, 20, and 10 μm, respectively. In Panel **b**, multiple irregularly shaped MoS_2 sheets and a rectangular strip of metal Au can be seen. The Au strip is used as label to mark the location of 1L MoS_2 areas to facilitate fast locating during STEM imaging. Panel **c** shows enlarged image covering the Au marker and the MoS_2 sheet containing 1L area, while Panel **d** focuses on the 1L MoS_2 area that is denoted by dotted lines.

12. Statistics on vacancy density of as-etched monolayers

Supplementary Fig. 12**a, b** shows typical high-angle annular dark-field STEM (HAADF-STEM) atomic images for local areas of the as-etched 1L MoS_2. In the HAADF imaging mode, the brightest dots and their adjacent slightly less bright ones correspond to the heavy Mo and light S atoms, respectively, as represented in Supplementary Fig. 12**a**. The lattice vacancies, i.e., missing atoms, would appear with a reduced brightness as compared to occupied sites, as indicated by the red arrow in Supplementary Fig. 12**b**. Supplementary Fig. 12**c** plots number of samples versus vacancy density and Gaussian distribution was used to fit the data, which reveals an average value of 1.3×10^{13} cm^{-2} with standard deviation of 0.6×10^{13} cm^{-2}, respectively.

13. Estimation of uncertainty for top-view elemental mapping

The uncertainty for EDS elemental mapping for the top-view samples becomes higher than that for the cross-sectional samples because of the nature of 1L thickness and fewer numbers of atoms involved for analysis. EDS signals for various elements including Mo, S, Al, Mg, Ar, Sc, Yb and Gd were carefully collected and some are shown in Supplementary Fig. 13**b–f**. The overall counts collected from detector are summarized and compared in Panel **g**. Among them, the elements Mo and S come from the sample and exhibit the strongest EDS signals in terms of the detector counts over the whole sampling area. The elements Mg and Ar are likely introduced by previous samples and their residues constitute the source of chamber contamination; they show less strong signals. Accordingly, the three selected rear-earth elements Sc, Yb, and Gd, which have never been directly introduced in the chamber and believed to be absent in the chamber, are used for calibrating the noise of imaging detection; they show the

weakest signals. The element Al, which is the one to be analyzed and shows signal intensity between Mg and Ar, can have two sources: sample and chamber contamination. In Panel **g**, we careful analyze the levels of chamber contamination and detection noise, as indicated by the blue and green bars, respectively. We estimate that the content of Al residues is within the level of chamber contamination. At the most, the trace of Al residues is about 5% if only ruling out the effect of detection noise.

14. Check of Al residues with XPS

We also performed XPS analysis to double check the content of Al residues. The result is quite similar to the EDS analysis above that no noticeable Al content is detected. As shown in Supplementary Fig. 14, the signal from Al 2*p* peak (around 74.5 eV) is at the level of noise in the XPS spectrum, proving that the Al residue is practically negligible. Note that two 100-nm Au pads were placed to mark the as-etched MoS_2 flake and also used as the reference for XPS analysis. The peak at 84.3 eV is from the excitation of the Au $4f_{7/2}$ level.

15. An alternative method for defect engineering

To further verify the crucial role of surface defects played in diffusion enhancement, we also tried other methods in introducing surface defects onto MoS_2. Supplementary Fig. 15 show the results for using pre-annealing as the defect engineering. In Panel **a**, an exfoliated MoS_2 sheet was pre-treated at 400 °C for 1 h before Al deposition. Then, an Al strip was deposited onto the sheet, followed by thermal diffusion at 250 °C for 0.5 h (Panel **b**) and acid wash (Panel **c**). The result shows that a monolayer of MoS_2 was removed successfully and homogeneously, indicating that diffusion is indeed enhanced in defective lattices. Hence, pre-annealing can be used as an alternative method for interfacial defect engineering.

16. Universality of the etching method

Besides MoS_2, we also applied this method to different TMDC sheets including WS_2 and WSe_2 to check its universality of the layer-by-layer processing. Supplementary Fig. 16 shows the Raman and photoluminescent spectra for the locally 0C, 1C and 2C processed WS_2 and WSe_2 trilayer sheets. After such consecutive thinning, local areas with thickness of 1L, 2L and 3L can be obtained accordingly. Panel **a** shows the Raman spectra collected for the processed local WS_2 areas whose Raman

characteristics resemble those of MoS_2; the distance of E_{2g}^1 (~355 cm^{-1}) and A_{1g} (~418 cm^{-1}) modes varies with the number of layers, i.e., etching cycles. The values of peak distance versus etching cycle are summarized in Panel **b**. Panel **c** shows the Raman spectra for the processed local WSe_2 areas. Their E_{2g}^1 (~251 cm^{-1}) and A_{1g} (~249 cm^{-1}) modes coincide practically with each other and peak distance cannot distinguish the information of number of layers any more. It was reported that the second-order mode 2LA(M) around 260 cm^{-1} gradually emerges as WSe_2 is thinned down from 3L to 1L. Also, the B_{2g}^1 peak of WSe_2 shows alternative odd-even dependency with thickness decreasing, which is coincided with published result using 488 nm laser[2–4]. The unusual thickness dependence exhibited by the three modes corroborates that the digital layer-by-layer etching technique on TMDCs is applicable.

The PL characteristics of MoS_2 and WS_2 and WSe_2 were also added and listed as Panels **d-f**, respectively, showing a strong PL excitation due to the transition to direct band gap when the flakes are etched down to monolayer. All the three thickness dependent PL characteristics are inconsistent with the exfoliated counterparts (WS_2, WSe_2: [5]; MoS_2:[6].) reported in literature.

17. Electrical properties of as-etched MoS_2 on SiO_2/Si

Systematic electrical characterizations were carried out on the field-effect transistors consisted of as-etched MoS_2 fabricated on SiO_2/Si substrates, as a comparison with those etched by other methods and, also, as a reference to those supported by ultraclean h-BN substrates. In Supplementary Fig. 17**a, b**, the transfer curves of the 2L and 1L layers reveal average threshold voltage values at 10.5 and 9.1 V, which are translated into positive doping levels of 2.6 and 2.3 × 10^{12} cm^{-2}, respectively. The appearance of overall positive rather than negative doping effect, as expected from Al as electron donor, indicate that the adverse doping induced by the Al residues is practically negligible, being much weaker than the impacts from the heavily doped silicon gates and surface gaseous absorbates. In addition, both devices exhibit a high I_{on}/I_{off} ratio of 10^8, suggesting that the semiconducting nature is well preserved in the as-etched layers.

The average two-probe carrier mobility (μ) of the two devices are calculated to be 16 and 31 $cm^2V^{-1}s^{-1}$, respectively. These values are comparable to these of exfoliated samples with similar thickness values (Supplementary Fig. 17**c**), thus it can be inferred that the acid wash can remove nearly all the Al residues and provide a clean and fresh

surface for the as-etched samples. In Supplementary Fig. 17**c**, we also compared the μ values of as-etched samples treated with various etching techniques, including thermal[7], laser[8,9], and plasma[10–12]. Apparently, by implementing the concept of selective etching, our etching method yields samples with the highest electronic quality among the in-situ etching techniques established so far. In other word, our method is a truly non-invasive surface etching technique that can preserve the electronic quality to the most.

Electrical characterizations at variable temperature from 10 to 300 K were carried out on an as-etched 2L device to evaluate the impact of extra lattice defects on the transport mechanism in MoS_2. Supplementary Fig. 17**d** shows its transfer curves at different temperatures. The linear plots of I_{ds} in the inset highlight that the metal-insulator transition point of the device is around 37 V. The presence of metal-insulator transition at high carrier concentration confirms again the high crystallinity is preserved after etching. We also extracted the hopping characteristic temperature T_0 through the 2D Mott variable-range hopping (VRH) equation. The extracted values of T_0 is plotted versus V_g in the inset of Supplementary Fig. 17**e**. T_0 ranges from 1.1×10^3 to 4.9×10^5 K, comparable to the values reported in exfoliated MoS_2 on SiO_2/Si substrates[13], which indicates that the extrinsic disorder introduced by Al residues is insignificant.

In Supplementary Fig. 17**f**, we plot μ versus T to further discern the effect of carrier scattering from Al residues. At high T, μ follows a power law with T ($\mu \propto T^{-\gamma}$ with γ~0.71) in the log-log plot. At low T, μ becomes saturated at ~70 $cm^2V^{-1}s^{-1}$. Both the values of μ and γ are much lower than the counterparts supported by ultraclean h-BN, indicating that that charge impurities at the SiO_2 surfaces play important role. Hence, ultraclean dielectric interfaces are critical for achieving the intrinsic performance of as-etched TMDCs.

References

1. Mehrer, H. *Diffusion in solids: fundamentals, methods, materials, diffusion-controlled processes* vol. 155 (Springer, Berlin, Heidelberg, 2007).

2. Luo, X. *et al.* Effects of lower symmetry and dimensionality on Raman spectra in two-dimensional WSe_2. *Phys. Rev. B* **88** (2013).

3. Terrones, H. *et al.* New first order Raman-active modes in few layered transition metal dichalcogenides. *Sci. Rep.* **4** (2014).

4. Tonndorf, P. *et al.* Photoluminescence emission and Raman response of monolayer MoS_2, $MoSe_2$, and WSe_2. *Opt. Express* **21**, 4908–4916 (2013).

5. Zhao, W. *et al.* Lattice dynamics in mono- and few-layer sheets of WS_2 and WSe_2. *Nanoscale* **5**, 9677–9683 (2013).

6. Mak, K. F., Lee, C., Hone, J., Shan, J. & Heinz, T. F. Atomically thin MoS_2: A new direct-gap semiconductor. *Phys. Rev. Lett.* **105**, 136805 (2010).

7. Wu, J. *et al.* Layer thinning and etching of mechanically exfoliated MoS_2 nanosheets by thermal annealing in air. *Small* **9**, 3314–3319 (2013).

8. Sunamura, K., Page, T. R., Yoshida, K., Yano, T.-A. & Hayamizu, Y. Laser-induced electrochemical thinning of MoS_2. *J. Mater. Chem. C* **4**, 3268–3273 (2016).

9. Castellanos-Gomez, A. *et al.* Laser-thinning of MoS_2: On demand generation of a single-layer semiconductor. *Nano Lett.* **12**, 3187–3192 (2012).

10. Kim, K. S. *et al.* Ultrasensitive MoS_2 photodetector by serial nano-bridge multi-heterojunction. *Nat. Commun.* **10**, 4701 (2019).

11. Kim, K. S. *et al.* Atomic layer etching mechanism of MoS_2 for nanodevices. *ACS Appl. Mater. Interfaces* **9**, 11967–11976 (2017).

12. Liu, Y. *et al.* Layer-by-layer thinning of MoS_2 by plasma. *ACS Nano* **7**, 4202–4209 (2013).

13. Jariwala, D. *et al.* Band-like transport in high mobility unencapsulated single-layer MoS_2 transistors. *Appl. Phys. Lett.* **102**, 173107 (2013).

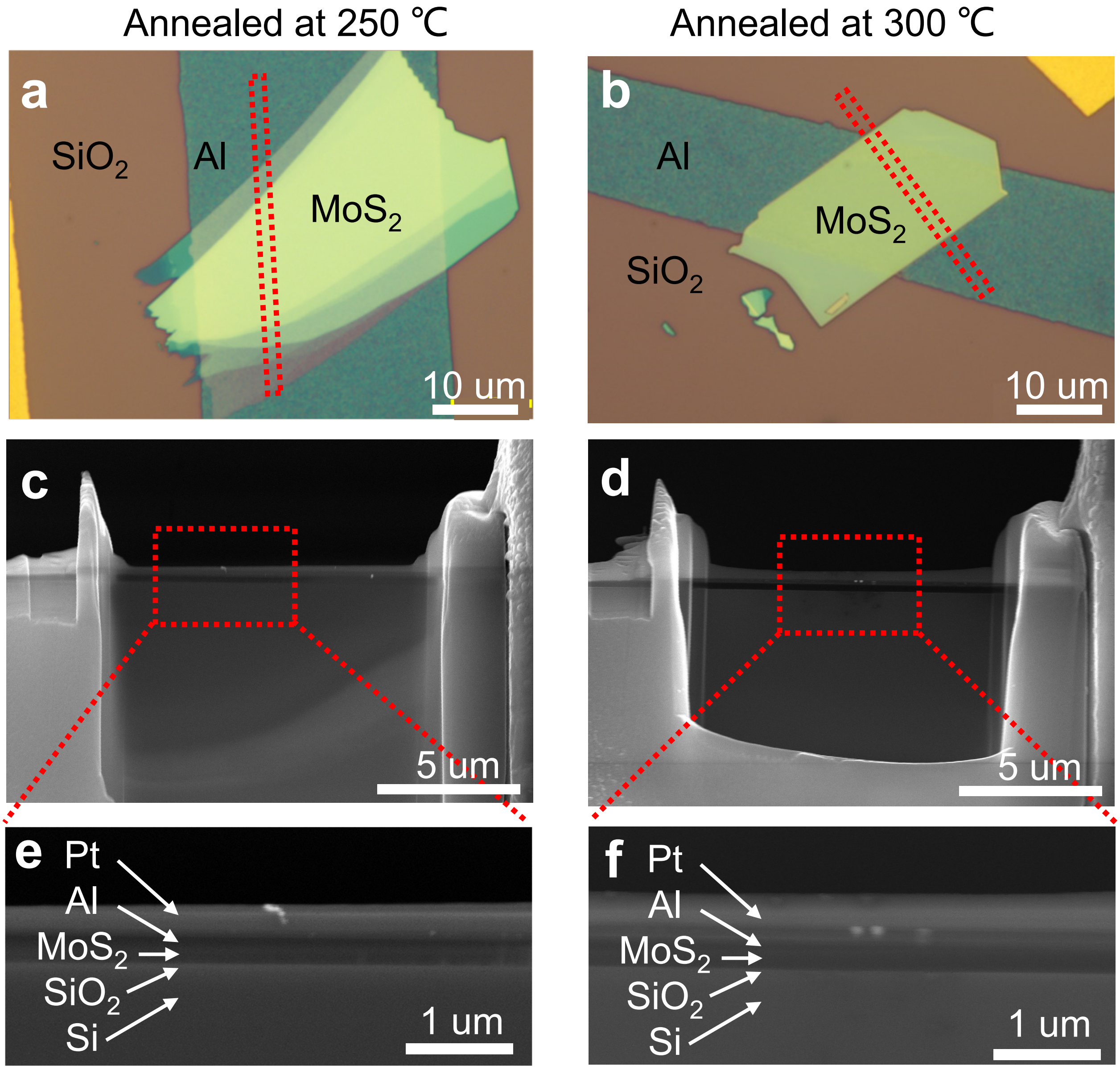

Supplementary Figure 1. Preparation of cross-sectional STEM specimens by FIB milling. a and **b,** Typical optical images for Al/MoS_2 stacks on SiO_2/Si substrates after one-hour thermal annealing at 250 and 300 °C, respectively. **c** and **d,** Corresponding SEM images for the cross-sectional slices after FIB milling. **e** and **f,** Enlarged images for selected areas shown in **c** and **d.** Note that Pt capping layers were deposited before FIB milling to protect samples from local deformation and ionizing radiation.

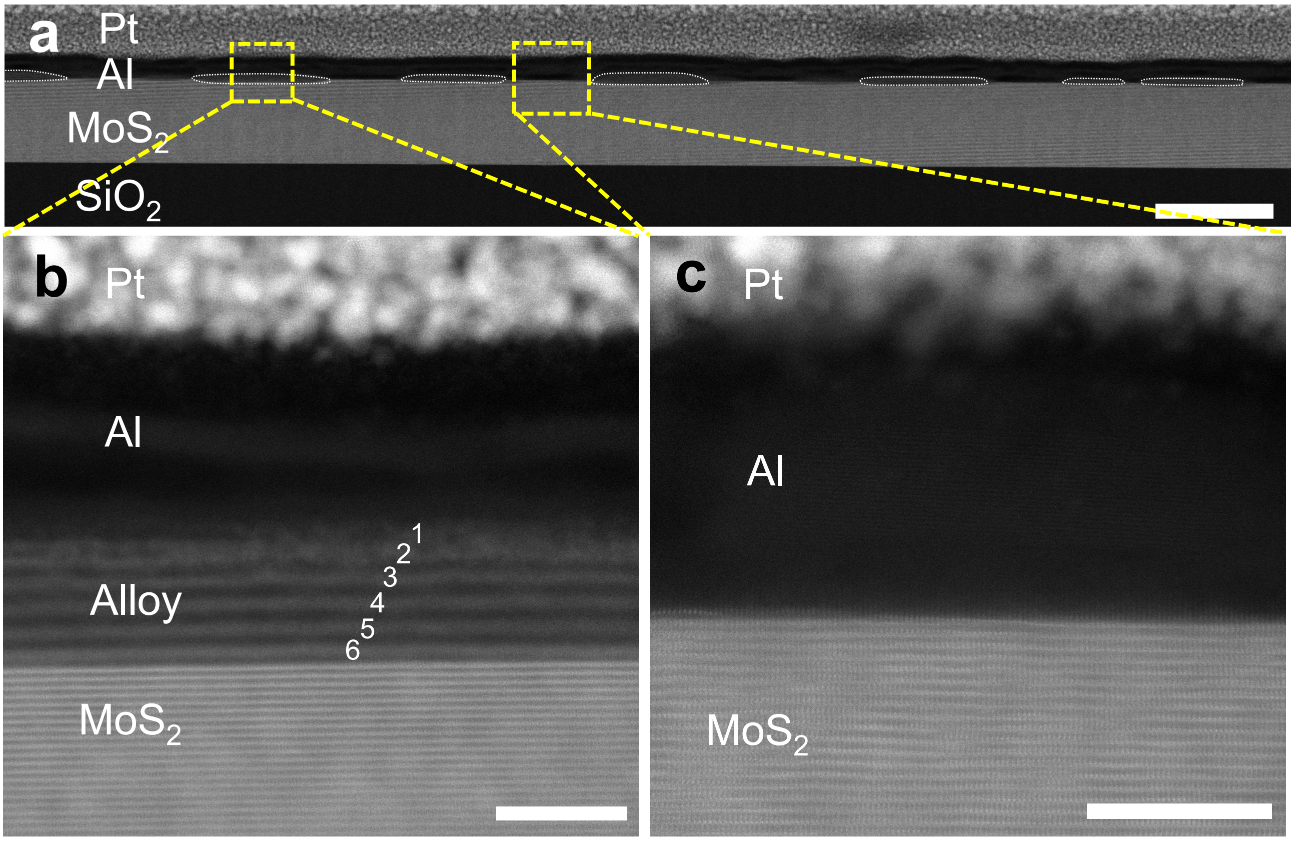

Supplementary Figure 2. High-resolution cross-sectional STEM images. a, Large scale HAADF image for a stack of Al and pristine MoS_2 annealed at 300 °C for 1 h. Note that the Pt capping layer is employed for sample protection. The alloy regions arising from interlayer diffusion between Al and MoS_2 are circled by white dotted lines. The diffused and undiffused areas occur with nearly equivalent probabilities. Scale bar, 100 nm. **b,** Zoom-in HAADF image for the center of an alloy region, where 6 layers of MoS_2 are delaminated by Al atoms as indicated by the numbers from 1 to 6. Among the 6 layers, the top 2 layers overlap with each other and become unclear, but the bottom 4 layers still keep the parallel characteristic spatially, which is inherited from their pristine lattice structure. **c,** Zoom-in HAADF image for an undiffused area. No obvious Al/MoS_2 interlayer diffusion is observed. Scale bars in panels **b** and **c** are 10 nm.

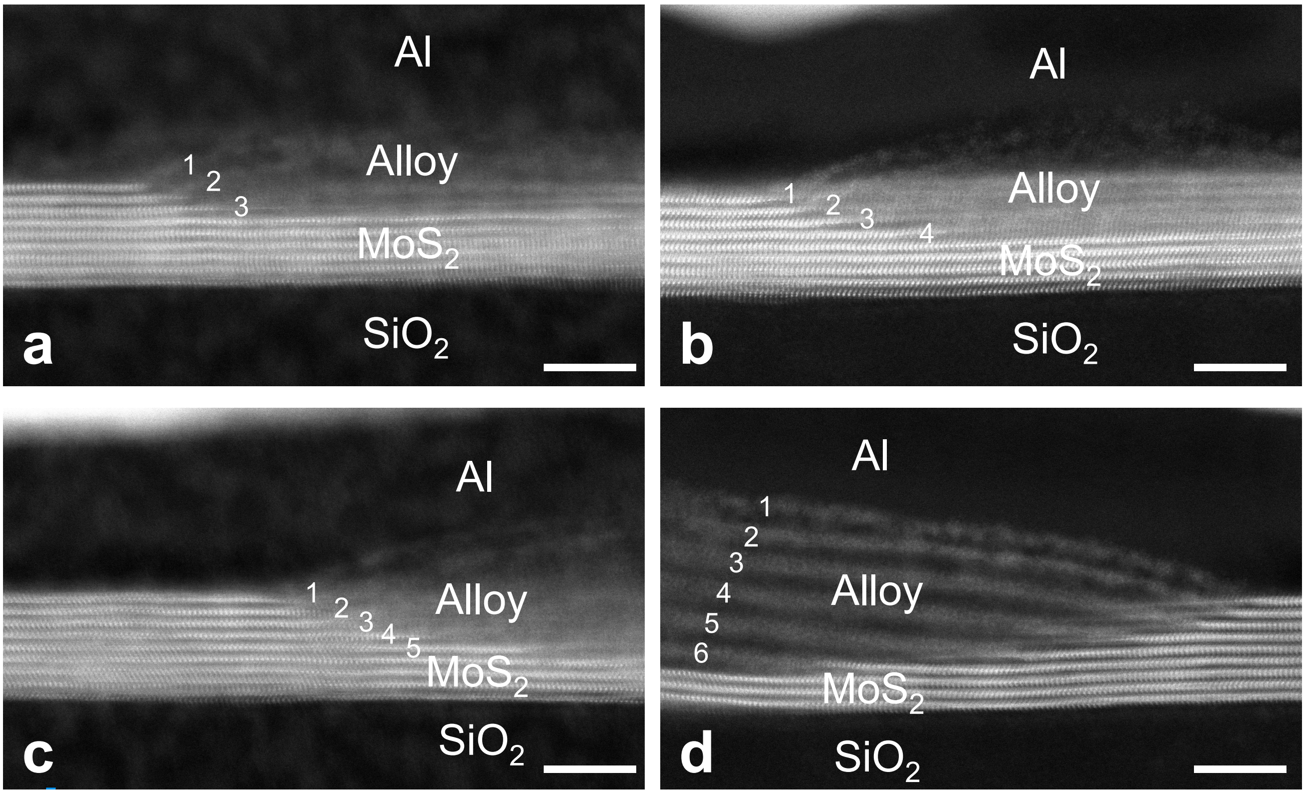

Supplementary Figure 3. Typical cross-sectional HR-STEM images taken around the beak-shaped alloy edges (droplets) to show the variation in diffusion depth under annealing condition of 300 °C for 1 h. Diffusion depths are 3L, 4L, 5L, 6L from Panel **a** to **d**, respectively. Scale bar, 5 nm.

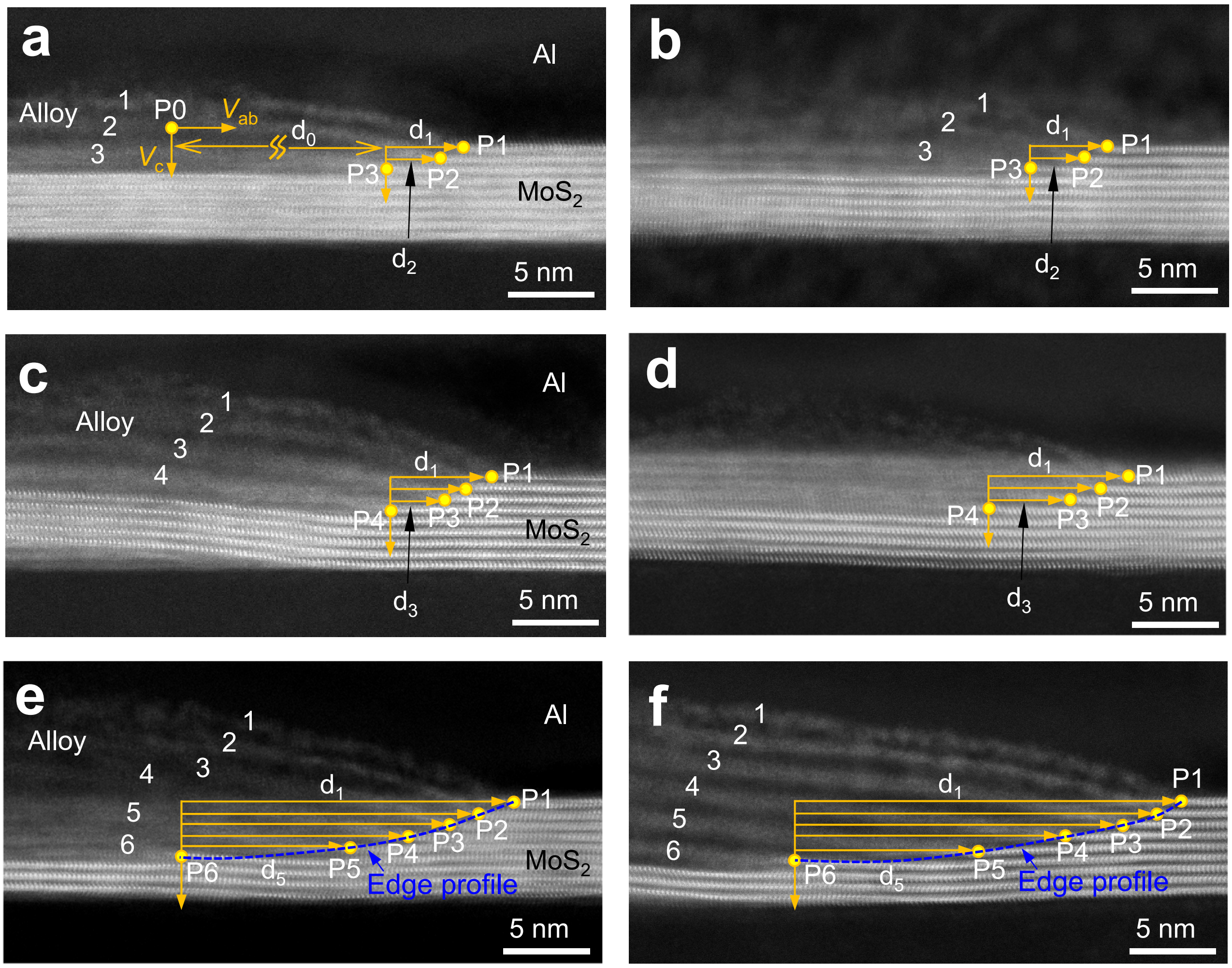

Supplementary Figure 4. Analysis on the length of lateral diffusion from the profiles of the beak-shaped alloy edges in the Al/MoS_2 stacks annealed under condition of 300 °C, 1 h. a-f, Typical cross-sectional HR-STEM images showing varied diffusion depths of 3L (**a** and **b**), 4L (**c** and **d**) and 6L (**e** and **f**), respectively. The layers of Al/MoS_2 alloy are indicated by the number n with n = 1, 2, 3…, representing the numbers of alloy layer counted from the topmost to the bottom. Accordingly, the critical diffusion terminals on the edge profiles are labeled as Pn with n = 1, 2, 3… As an example, the edge profiles in **e** and **f** are labeled by blue dotted lines.

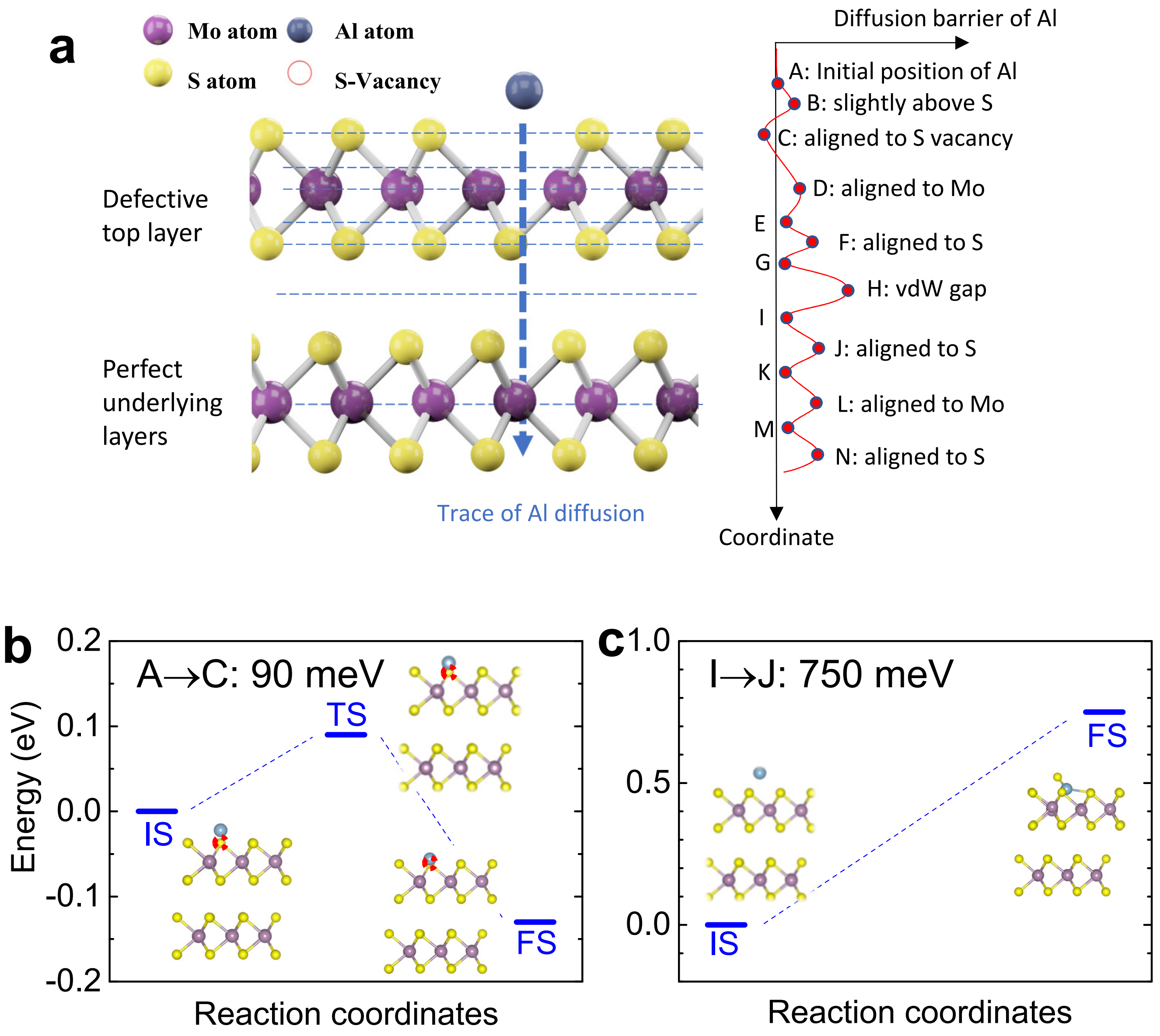

Supplementary Figure 5. Estimated diffusion energies for Al atoms into defective and perfect MoS_2 lattices. a, Schematic diagram for an Al atom passing through the defective top and perfect underlying MoS_2 layers, respectively. On the right is schematically plotted the potential curve at different coordinates of the MoS_2 lattice. **b** and **c**, Diffusion energy curves calculated by density functional theory for Al atoms into the defective (90 meV) and perfect (750 meV) MoS_2 layers, respectively.

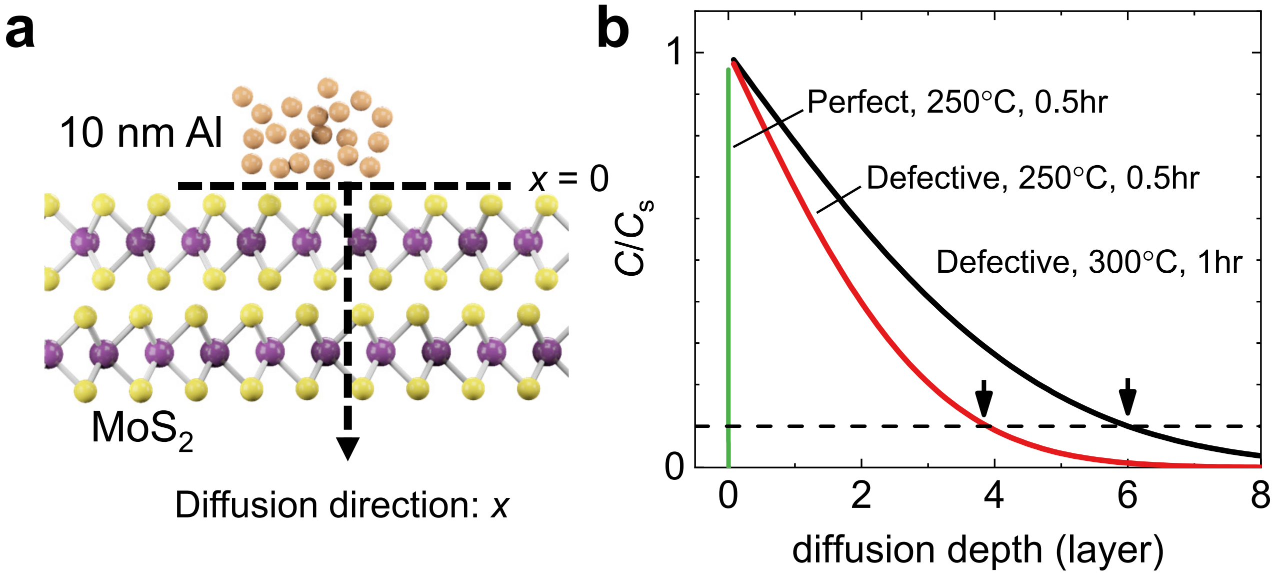

Supplementary Figure 6 . A simplified estimation on the diffusion distribution of Al atoms along the diffusion direction with a continuum model by neglecting the complicated profile of diffusion barrier at different coordinates of the MoS_2 lattices. a, Schematic diagram for the diffusion flux of Al atoms into a MoS_2 lattice. The diffusion direction is assigned as *x*. **b,** Calculated normalized concentration of Al element as a function of diffusion depth for three different combinations of sample quality and annealing condition. The criterion of diffusion cutoff is taken as 0.1 C_s. Hence, the diffusion depth of Al into defective MoS_2 under the annealing condition of 300 °C and 1 h is about 6 layers and is reduced to about 4 layers under the condition of 250 °C and 0.5 h. For a perfect MoS_2 lattice, the calculated diffusion depth is practically zero under the condition of 250 °C and 0.5 h.

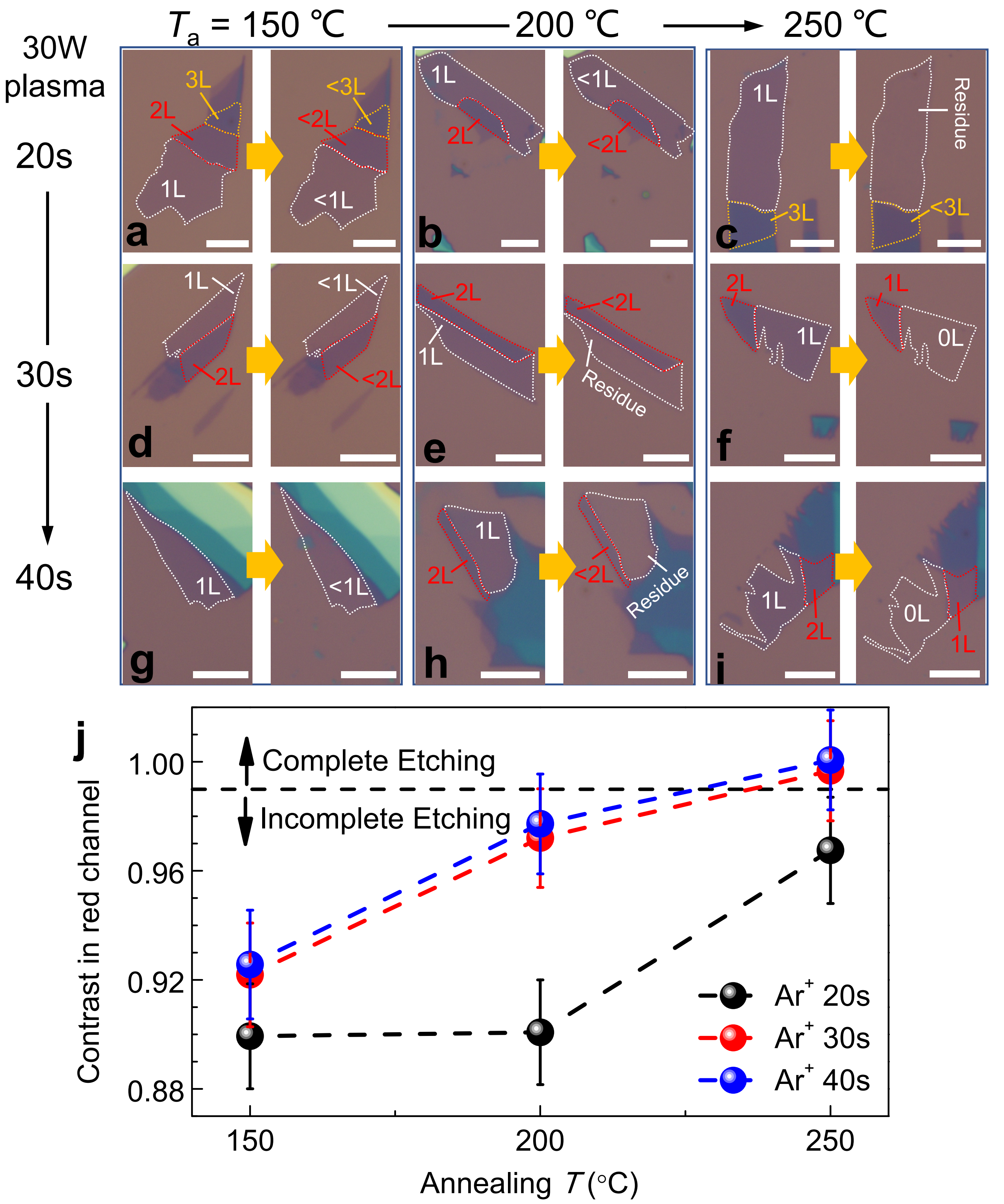

Supplementary Figure 7. Cross-over test on the etching results under different pretreatment conditions with the duration of plasma irradiation (30 W) varying from 20 to 40 s and thermal annealing T_a changing from 150 to 250 ℃. a-i, Optical images taken before and after etching for checking the residues at varied conditions. Scale bars, 5 μm. **j**, Image contrast of the etched areas with respect to nearby substrate areas, which can be used to scientifically judge the etching completeness. Standard deviations are used as error bars. The criterion is set as 0.99 in contrast for a completer etching process without invisible residues.

Conditions of Ar plasma: ICP power = 30 W, CCP power = 0 W.

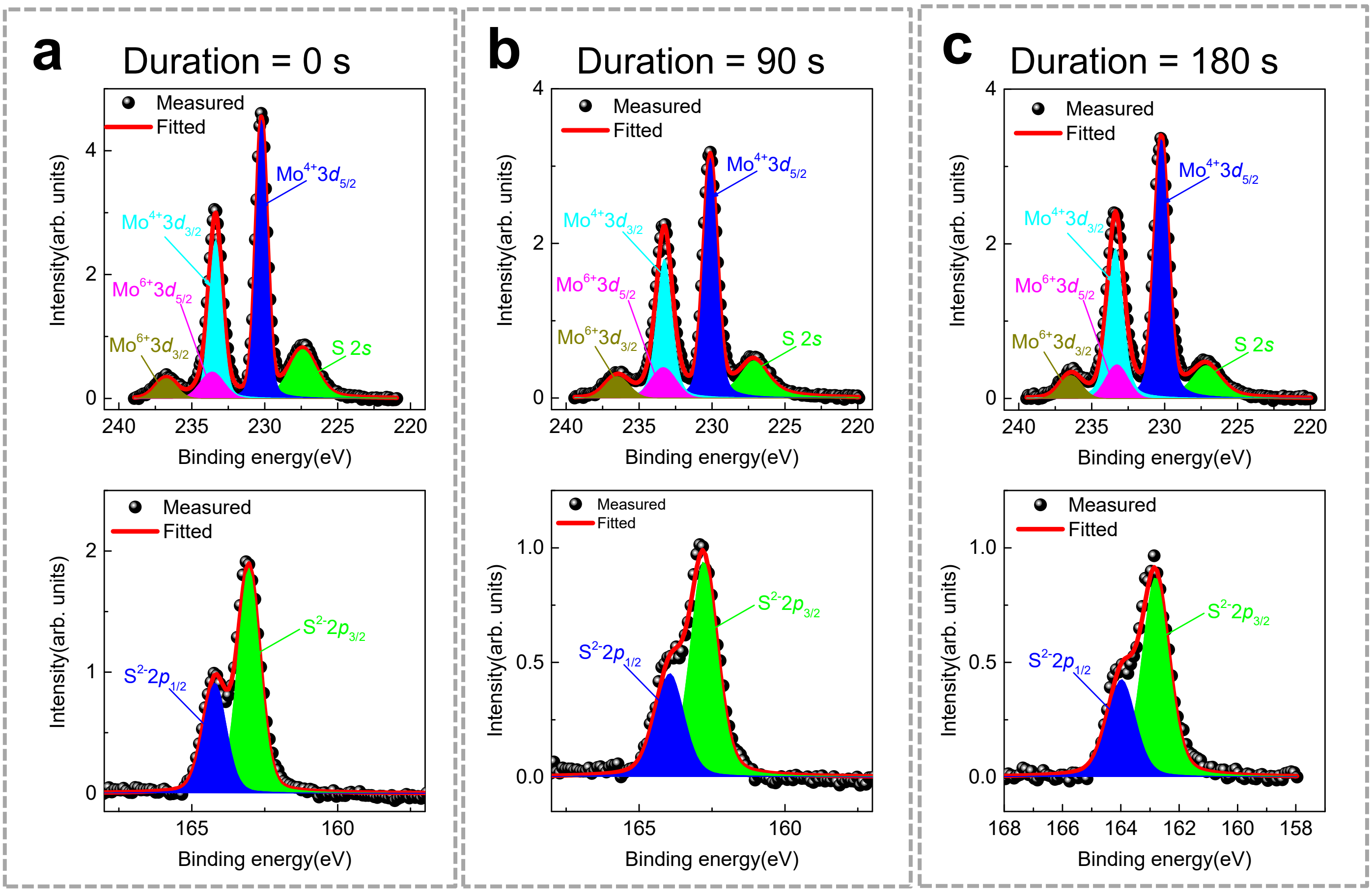

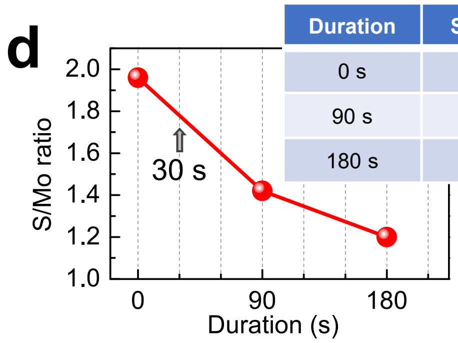

Duration	S/Mo ratio
0 s	1.96
90 s	1.42
180 s	1.20

e

Duration	S/Mo ratio	S atoms removed (%)	Density of S atom (10^{15} cm^{-2})
0 s	1.96	0	2.36
30 s	1.78	9.2	2.19
90 s	1.42	27.6	1.71

Supplementary Figure 8. XPS spectra and fitting results for S/Mo ratio in samples with varied durations of Ar plasma irradiation.

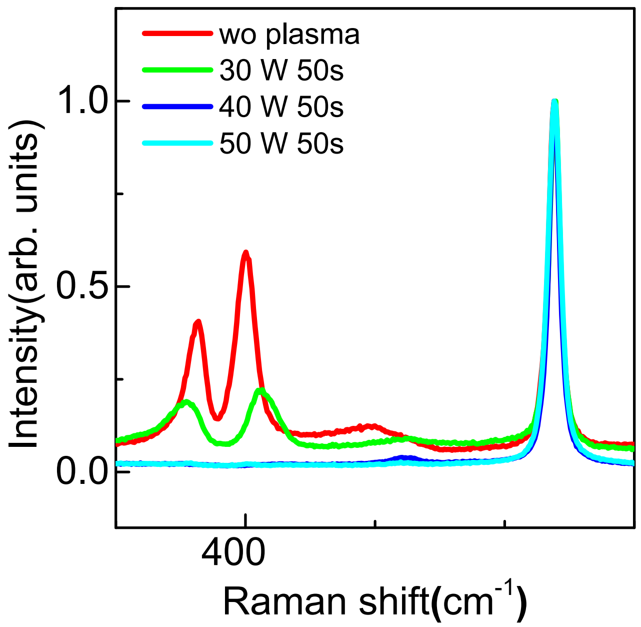

Supplementary Figure 9. Raman spectra for pristine and irradiated monolayer MoS_2 under different ICP powers of Ar plasma.

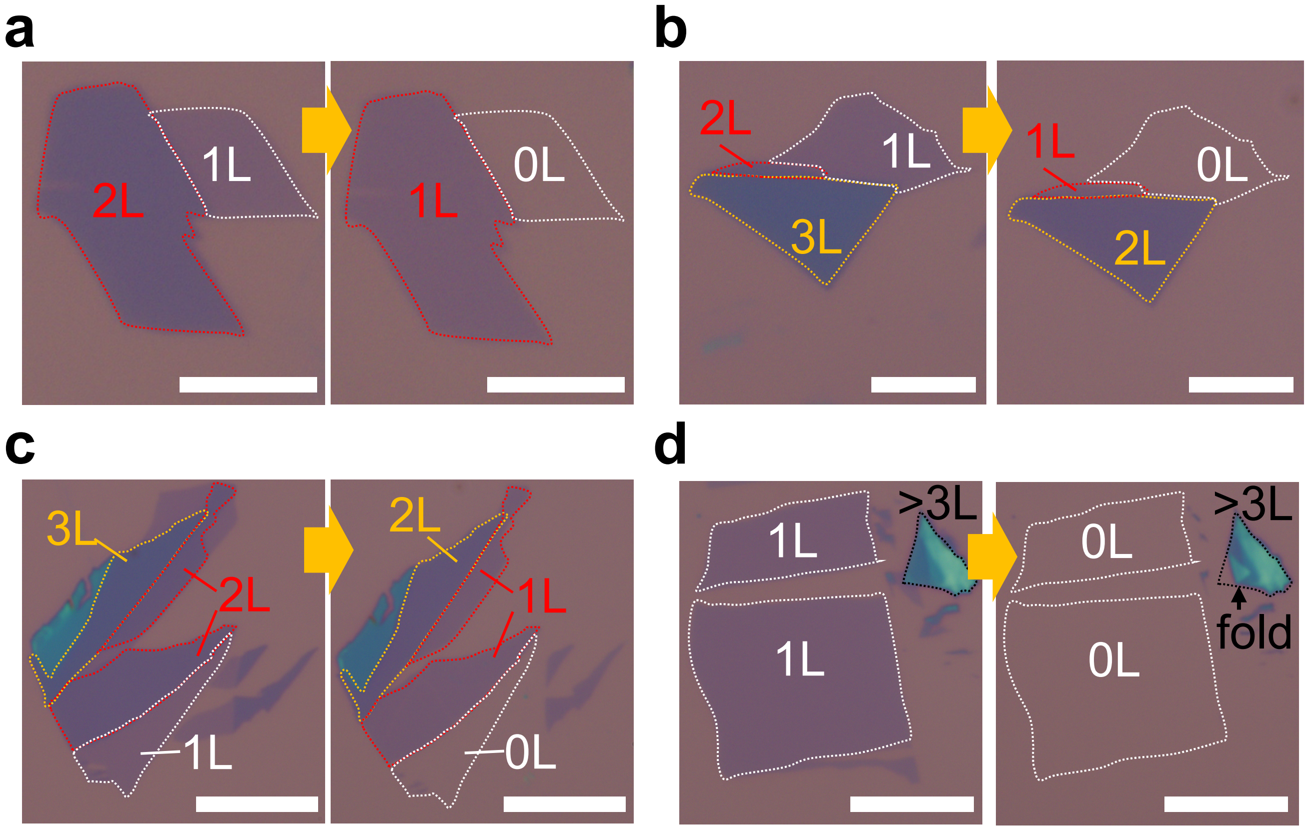

Supplementary Figure 10. Etching tests by using various acid and basic solutions. a, sulfuric acid; **b,** phosphoric acid; **c,** oxalic acid; **d,** alkalic TMAH solution. The local MoS_2 areas are all labeled by dash line in different colors. Under appropriated pretreatment condition, all tested four solutions above are effective. Scale bar: 5 μm in **a, b**; 10 μm in **c, d**.

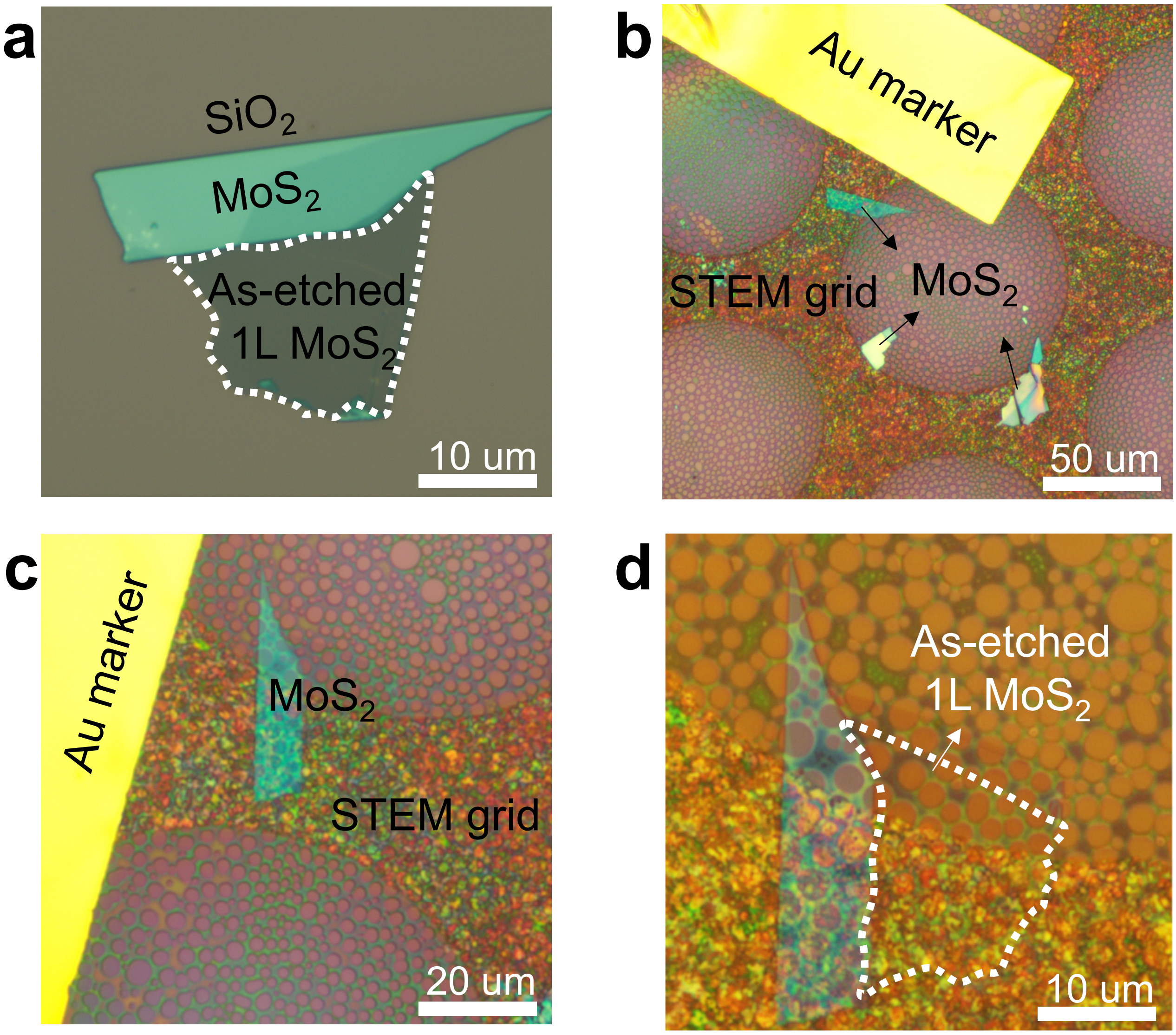

Supplementary Figure 11. Typical optical images for as-etched 1L MoS_2 supported on STEM grids as top-view specimen. a, As-etched 1L MoS_2 on SiO_2/Si substrates. **b–d**, Corresponding images for the specimen after transferring onto STEM grid at varied magnification ratios. A gold strip is used as maker to indicate the specimen location.

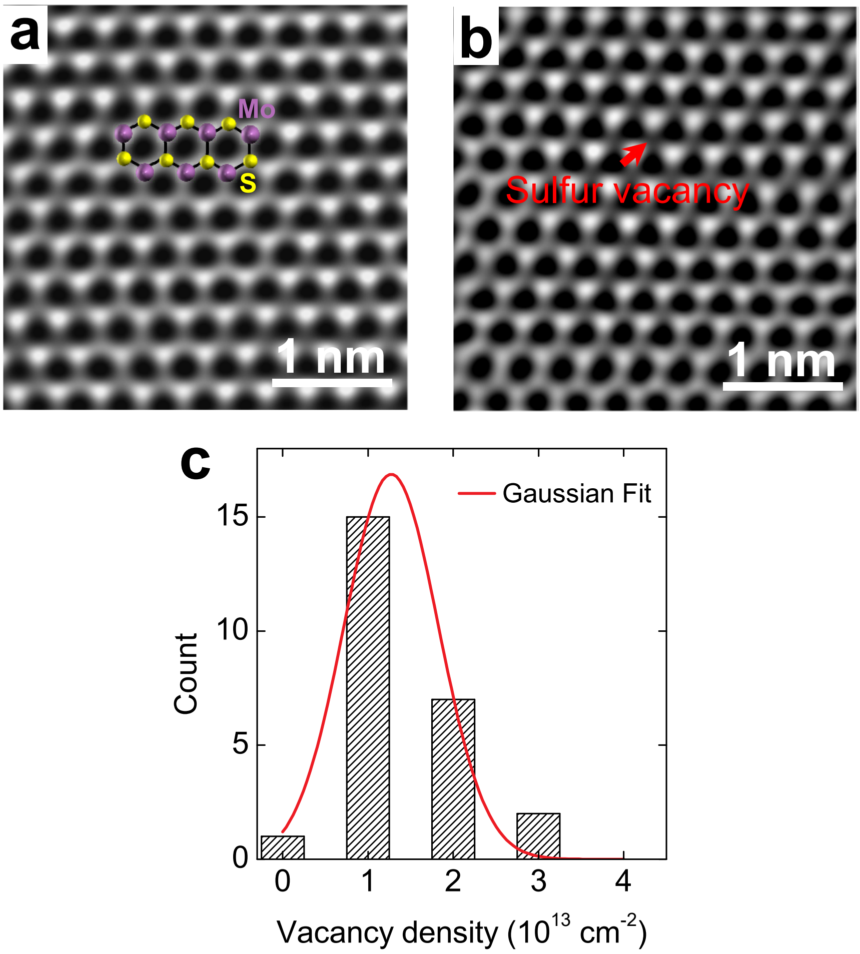

Supplementary Figure 12. Atomically resolved HR-STEM images for as-etched MoS_2 and statistics on vacancy density. a and **b,** Typical top-view HAADF images for different local areas of an as-etched monolayer, where the most and less bright dots represent the Mo and S atoms, respectively, as labeled by the purple and yellow balls in **a**. A sulfur vacancy can be seen in **b**, as indicated by red arrow. **c**, Histogram and corresponding Gaussian fit on the vacancy density from 25 local areas. The average value and standard deviation of the Gaussian fit are 1.3 and 0.6 $\times 10^{13}$ cm^{-2}, respectively.

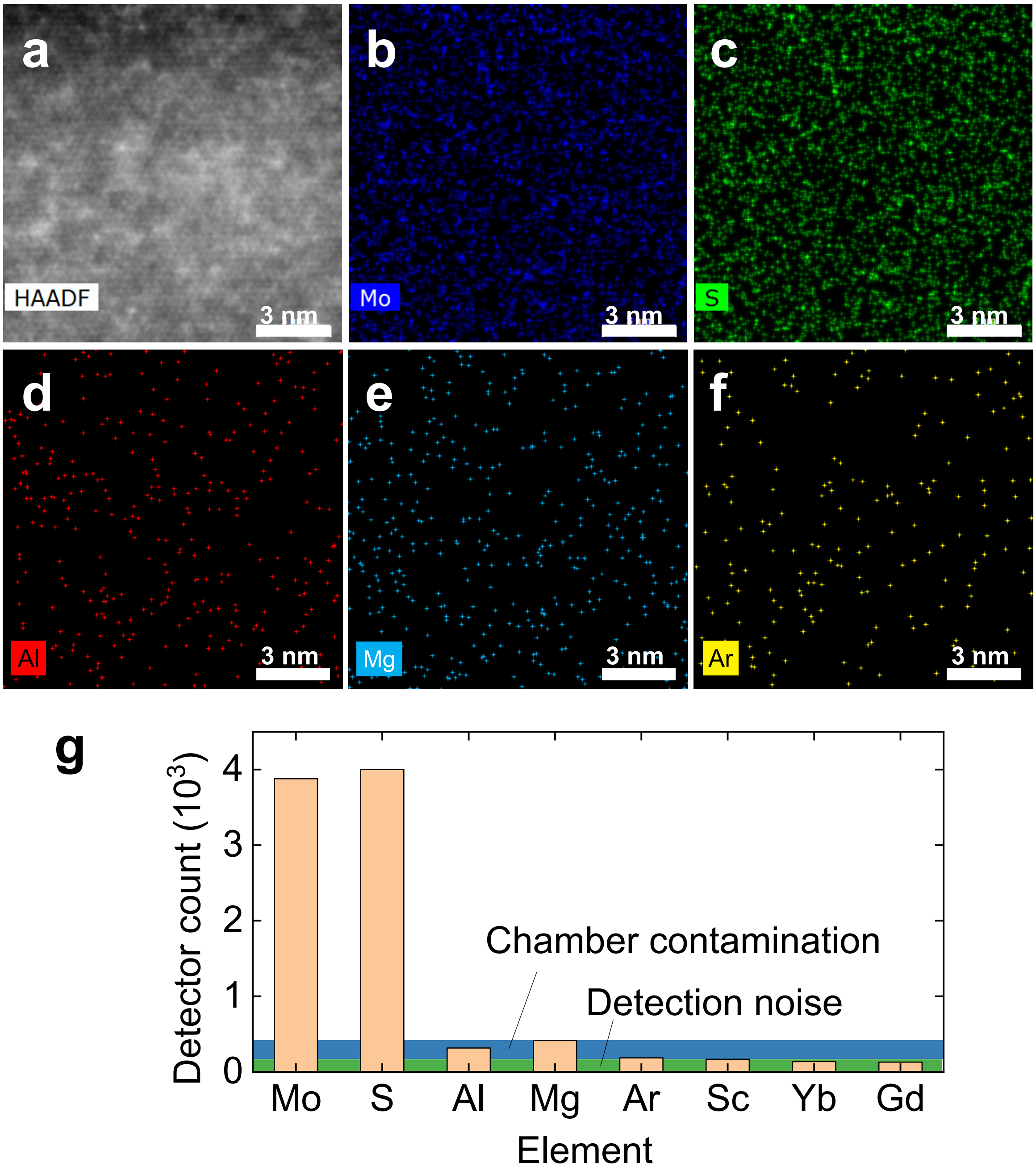

Supplementary Figure 13. Estimation of uncertainty for the top-view EDS elemental mapping. a, HAADF image of the area for elemental analyses. **b-f**, EDS mappings for the Mo, S, Al, Mg and Ar elements, respectively. **g,** Statistics for the contents of varied elements. Among them, the detector counts of the rear-earth elements Sc, Yb, and Gd, which have been never directly introduced in the chamber and are believed to be absent in the chamber, are used for calibrating CCD detection noise, and Mg and Ar are likely introduced into chamber from previous samples and hence used for calibrating chamber contamination.

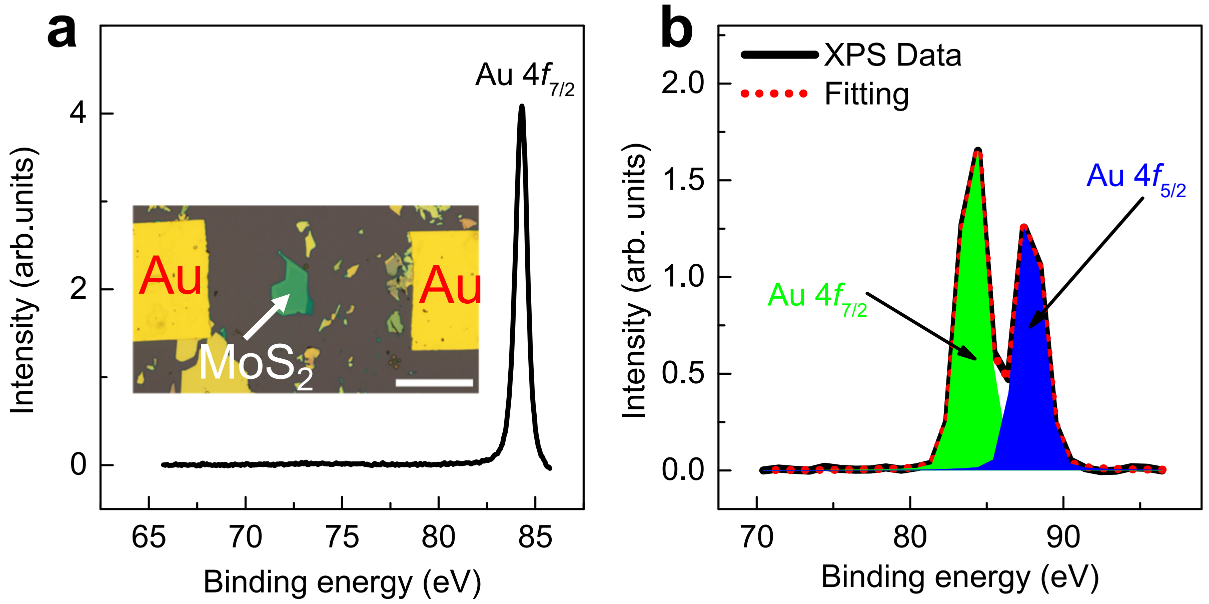

Supplementary Figure 14. XPS characteristics of as-etched MoS_2 for double check on the trace of Al residues a, XPS spectrum taken around the Al 2*p* peak where no significant signal was detected from Al residues. The peak at 84.3 eV is assigned to Au $4f_{7/2}$. Inset: optical image of a MoS_2 sample used in XPS. Two Au strips were placed as marker to locate the position of MoS_2. Scale bar, 50 μm. **b,** Spectrum with extended energy range in which the doublet from the Au 4*f* level can be fully observed and the signals of Al remain elusive.

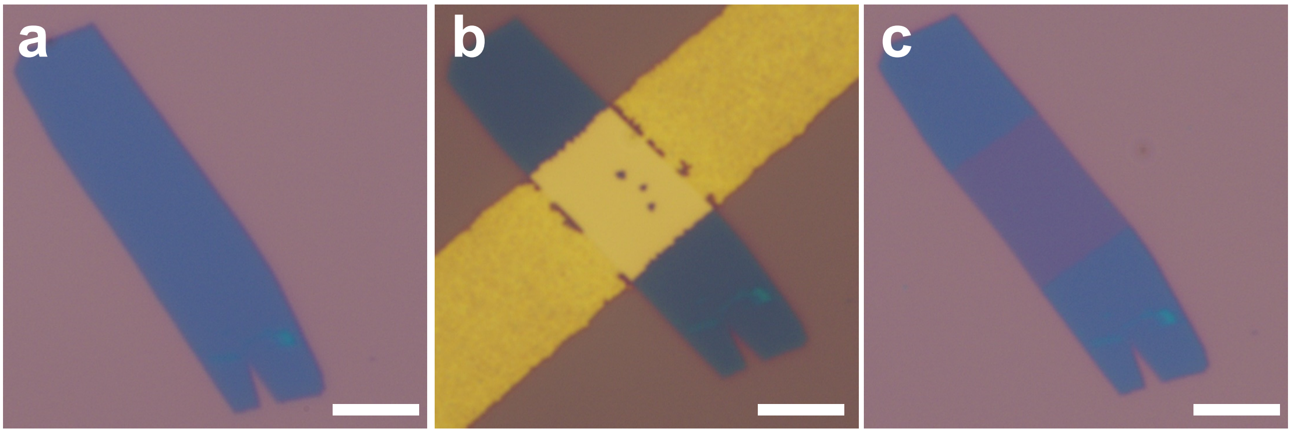

Supplementary Figure 15. An alternative method for defect engineering via thermal decomposition. a, Optical image for a typical MoS_2 flake under pre-annealing condition of 400 °C, 1 h to form surficial vacancies before Al deposition. **b,** After deposition of an Al strip and thermal annealing at 250 °C for 0.5 h to render Al diffusion. **c,** After acid wash where a monolayer is removed from the center. Scale bar, 5 μm.

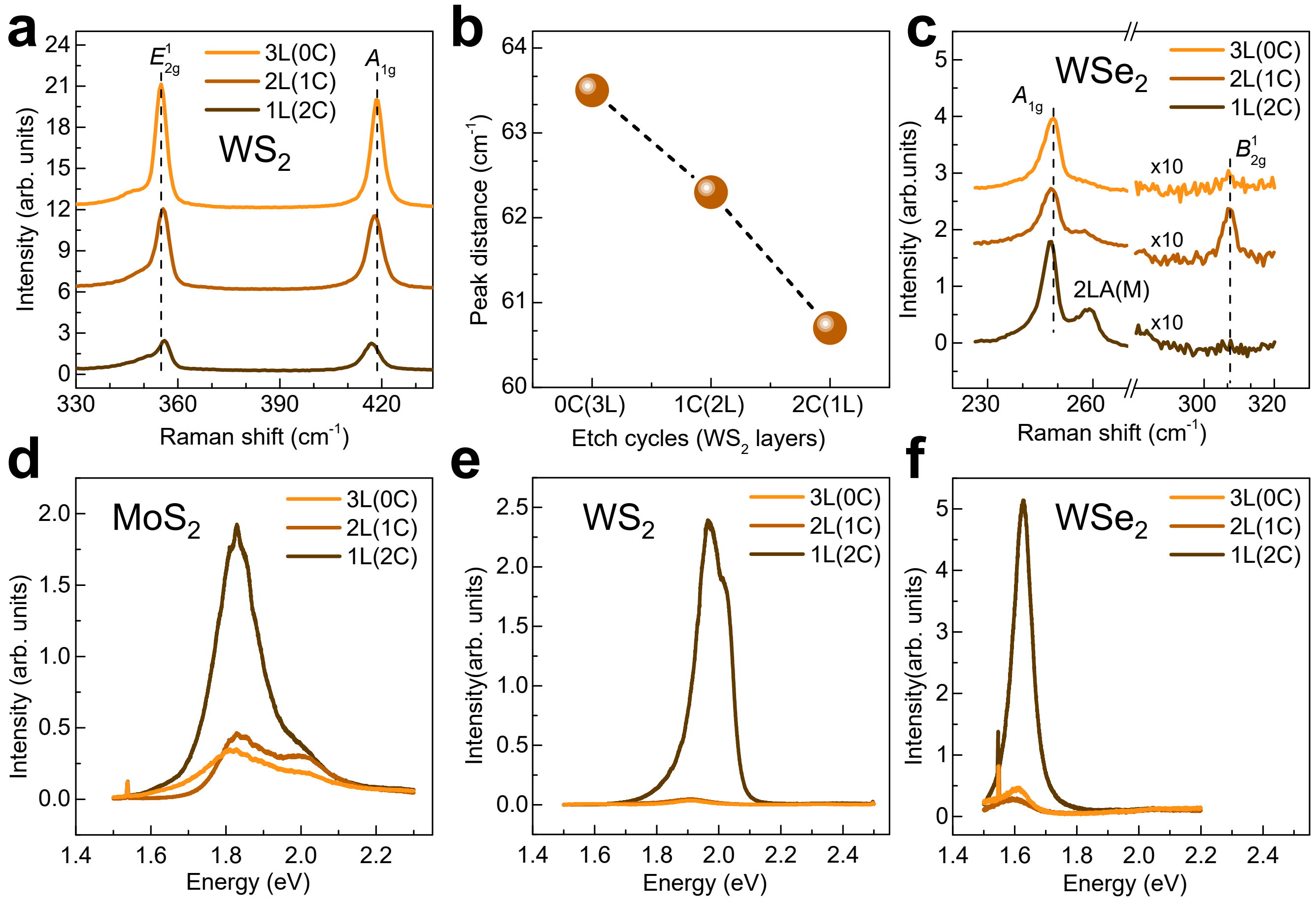

Supplementary Figure 16. Raman and photoluminescent (PL) spectra taken at different cycles for twice-etched TMDCs, including MoS_2, WS_2 and WSe_2. a, Raman spectra for local areas of pristine (0C), one-cycle (1C) and two-cycle (2C) etched WS_2 (from top to bottom). **b,** Distance between $E_{2g}{}^1$ and A_{1g} modes versus etching cycles (i.e., number of WS_2 layers). The variation of peak distance confirms the applicability of precise layer-by-layer etching on WS_2. **c,** Raman spectra for the corresponding 0C, 1C, and 2C etched WSe2 (from top to bottom). The red shift of A_{1g} mode and the emergence of 2LA(M) mode with the decrease in the number of layers suggest the accurate thickness information of 1L, 2L and 3L areas. The $B_{2g}{}^1$ mode becomes noticeable only in the flakes with odd number of layers, which is consistent with the results reported in literature. **d-f,** Corresponding PL spectra for local areas of 0C, 1C, and 2C etched MoS_2 , WS_2 and WSe_2, respectively.

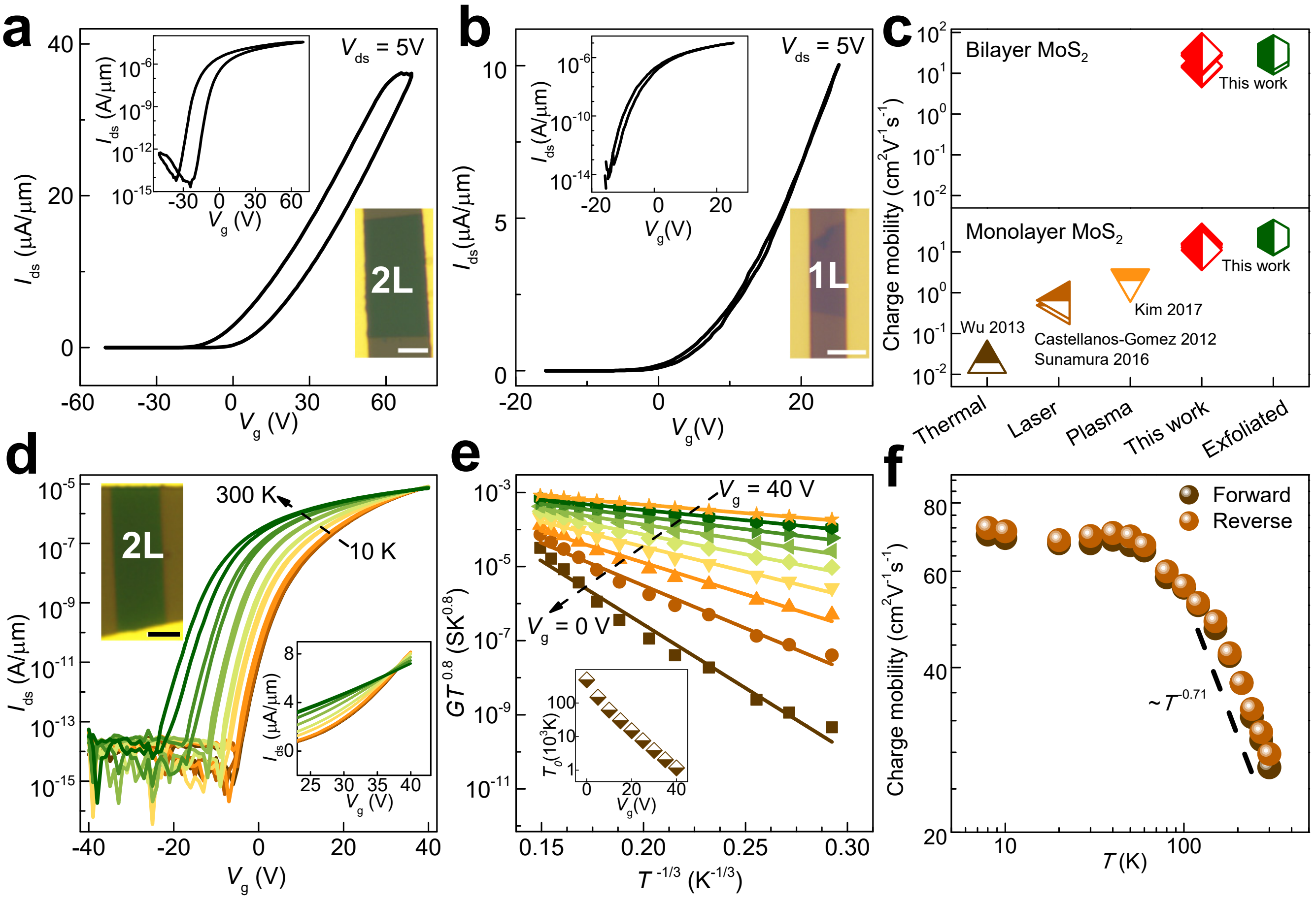

Supplementary Figure 17. Electrical properties for as-etched MoS_2 flakes on SiO_2/Si substrates. a-b, Two-probe transfer characteristics plotted in linear scale for the as-etched bilayer (2L) and monolayer (1L) MoS_2. Insets: the corresponding transfer curves in log scale and device images. **c,** Comparison of carrier mobilities of MoS_2 sheets prepared by our and other methods (thermal oxidation, plasma, laser and mechanical exfoliation). **d,** Variable temperature measurement of an as-etched 2L MoS_2 sheet. Transfer characteristics at different T values of 10 K, 30 K, 50 K, 80 K, 120 K, 180 K, 240 K and 300 K, respectively. The behavior of metal insulator transition (MIT) is highlighted in inset. **e,** Mott VRH model fitting for two-probe conductivity at various gate voltages. Inset: the fitted characteristic temperature T_0 versus gate voltage. The values are consistent with those reported in other exfoliated MoS_2 devices. **f,** Temperature-dependent field-effect mobility. At $T > 100$ K, the mobility follows $\mu \propto T^{-\gamma}$ with $\gamma = 0.71$. The black dashed line ($\sim T^{-0.71}$) is a guide to the eyes. Scale bar, 4 μm.
